# Fluid balance control in critically ill patients: results from POINCARE-2 stepped wedge cluster-randomized trial

**DOI:** 10.1186/s13054-023-04357-1

**Published:** 2023-02-21

**Authors:** Pierre-Edouard Bollaert, Alexandra Monnier, Francis Schneider, Laurent Argaud, Julio Badie, Claire Charpentier, Ferhat Meziani, Michel Bemer, Jean-Pierre Quenot, Marie Buzzi, Hervé Outin, Cédric Bruel, Laurent Ziegler, Sébastien Gibot, Jean-Marc Virion, Camille Alleyrat, Guillaume Louis, Nelly Agrinier

**Affiliations:** 1 Service de Réanimation Médicale, CHRU Nancy, Université de Lorraine, 54000 Nancy, France; 2 Service de Médecine Intensive-Réanimation Médicale, Nouvel Hôpital Civil, CHU Strasbourg, Université de Strasbourg, 67000 Strasbourg, France; 3 Service de Médecine Intensive-Réanimation, Hôpital de Hautepierre, CHU Strasbourg, INSERM U 1121, 67000 Strasbourg, France; 4 Service de Réanimation Médicale, Hospices civils de Lyon, Hôpital Edouard Herriot, 69000 Lyon, France; 5 Service de Réanimation Médicale, Hôpital Nord Franche-Comté, 90015 Belfort, France; 6 Service d’Anesthésie Réanimation Chirurgicale, CHRU Nancy, Université de Lorraine, 54000 Nancy, France; 7 Service de Réanimation Polyvalente, CHR Metz-Thionville, 57000 Thionville, France; 8 Service de Médecine Intensive-Réanimation, CHU Dijon-Bourgogne, 21000 Dijon, France; 9 CIC, Epidémiologie Clinique, CHRU Nancy, INSERM, Université de Lorraine, 54000 Nancy, France; 10 Université de Lorraine, Apemac, 54000 Nancy, France; 11 Service de Réanimation, CHI Poissy Saint-Germain, 78303 Poissy, France; 12 Service de Réanimation Polyvalente, Groupe Hospitalier Paris Saint-Joseph, 75000 Paris, France; 13 Service d’anesthésie Réanimation, CH Verdun, 55000 Verdun, France; 14 Service de Réanimation Polyvalente, CHR Metz-Thionville, 57000 Metz, France

**Keywords:** Critical care, Water-electrolyte balance, Clinical trial, Complex intervention

## Abstract

**Background:**

In critically ill patients, positive fluid balance is associated with excessive mortality. The POINCARE-2 trial aimed to assess the effectiveness of a fluid balance control strategy on mortality in critically ill patients.

**Methods:**

POINCARE-2 was a stepped wedge cluster open-label randomized controlled trial. We recruited critically ill patients in twelve volunteering intensive care units from nine French hospitals. Eligible patients were ≥ 18 years old, under mechanical ventilation, admitted to one of the 12 recruiting units for > 48 and ≤ 72 h, and had an expected length of stay after inclusion > 24 h. Recruitment started on May 2016 and ended on May 2019. Of 10,272 patients screened, 1361 met the inclusion criteria and 1353 completed follow-up. The POINCARE-2 strategy consisted of a daily weight-driven restriction of fluid intake, diuretics administration, and ultrafiltration in case of renal replacement therapy between Day 2 and Day 14 after admission. The primary outcome was 60-day all-cause mortality. We considered intention-to-treat analyses in cluster-randomized analyses (CRA) and in randomized before-and-after analyses (RBAA).

**Results:**

A total of 433 (643) patients in the strategy group and 472 (718) in the control group were included in the CRA (RBAA). In the CRA, mean (SD) age was 63.7 (14.1) versus 65.7 (14.3) years, and mean (SD) weight at admission was 78.5 (20.0) versus 79.4 (23.5) kg. A total of 129 (160) patients died in the strategy (control) group. Sixty-day mortality did not differ between groups [30.5%, 95% confidence interval (CI) 26.2–34.8 vs. 33.9%, 95% CI 29.6–38.2, *p* = 0.26]. Among safety outcomes, only hypernatremia was more frequent in the strategy group (5.3% vs. 2.3%, *p* = 0.01). The RBAA led to similar results.

**Conclusion:**

The POINCARE-2 conservative strategy did not reduce mortality in critically ill patients. However, due to open-label and stepped wedge design, intention-to-treat analyses might not reflect actual exposure to this strategy, and further analyses might be required before completely discarding it.

*Trial registration* POINCARE-2 trial was registered at ClinicalTrials.gov (NCT02765009). Registered 29 April 2016.

**Supplementary Information:**

The online version contains supplementary material available at 10.1186/s13054-023-04357-1.

## Background

Causes of positive fluid balance, observed in most critically ill patients [1], include reduced urine output subsequent to shock state, acute kidney injury, major surgical procedures, positive pressure mechanical ventilation [2], and simultaneous excessive fluid loading to maintain acceptable arterial pressure. Usually, this results in a positive cumulated fluid balance of about 4–10 L at the end of the first week of hospital stay [2–4]. Such a positive fluid balance is independently associated with impaired prognosis, notably mortality [5], in patients admitted to intensive care units (ICUs) for acute kidney injury [3, 6–8], acute respiratory distress syndrome (ARDS) [4, 9, 10], sepsis [10, 11], serious trauma [12], or high risk surgery [13]. Mechanisms underpinning the deleterious role of positive fluid balance in critically ill patients are unclear. Large volume fluid administration usually results in tissue edema and clinical manifestations of fluid overload [1, 14, 15]. Tissue edema can (i) impair metabolite diffusion, (ii) disrupt tissue and organ architecture, (iii) impede microcirculation, and (iv) reduce lymphatic flow; and may thus compromise cell–cell interactions [14, 15]. In addition, hypoalbuminemia can cause positive fluid balance by inducing negative oncotic pressure [1].

The effectiveness of interventions controlling fluid balance on survival remains unclear. Two randomized controlled trials of patients with ARDS showed that a strategy of fluid balance control (i.e., a conservative strategy) increased the number of mechanical ventilator-free days (MVFDs), and reduced ICU length of stay with no noticeable adverse effects [2, 16]. Adjunction of albumin administration to this strategy proved effective on oxygenation and hemodynamic stability in patients with hypoproteinaemia [17]. A recent pilot trial of patients with septic shock demonstrated that fluid restriction was feasible and well-tolerated, at least with respect to circulatory efficacy [18]. If found effective on hard endpoints and in a broader range of patients admitted to ICUs, an easy-to-implement strategy targeting fluid balance control would result in improved prognosis at limited cost. We hypothesized that a strategy targeting fluid balance control initiated 2 days after admission in a broad range of critically ill patients would result in an absolute reduction of all-cause mortality of 15% as compared with standard of care. We aimed to assess the effectiveness of a fluid balance control strategy on mortality in critically ill patients.

## Methods

### Study design

The protocol of POINCARE-2 (POids INtensive CARE 2) trial has been extensively detailed elsewhere [19]. Briefly, the POINCARE-2 trial consisted of a stepped wedge cluster open-label randomized trial involving 12 ICUs (i.e., clusters) of 9 French hospitals from June 2016 to May 2019. The *Comité de Protection des Personnes Est III,* Grand-Est, North-East France, has reviewed and approved POINCARE-2 trial (ID-RCB: 2015-A00662-47).

Because of (i) the complex design of the intervention (i.e., a strategy involving multiple health professionals and multiple steps of a decisional algorithm over 14 days requiring a learning phase), and (ii) the open-label design; and to address the potentially resulting contamination bias (i.e., delivery of the strategy in patients randomized to the control group), we opted for a cluster-randomized trial over an individual randomized trial. In addition, due to the amount of evidence suggesting the deleterious effect of fluid imbalance on survival and to the relatively easy-to-implement aspect of the strategy in daily ICU practice, we opted for a stepped wedge design to allow each participating ICU to eventually implement the strategy under scrutiny. A stepped wedge trial is a type of cluster-randomized trial where clusters (ICUs) recruit patients both in the control group during the first period (semester) of recruitment and in the strategy group during the last period (semester) of recruitment; and where the switch between control and strategy recruitment is set at a randomly assigned time (wedge).

**Fig. 1:**
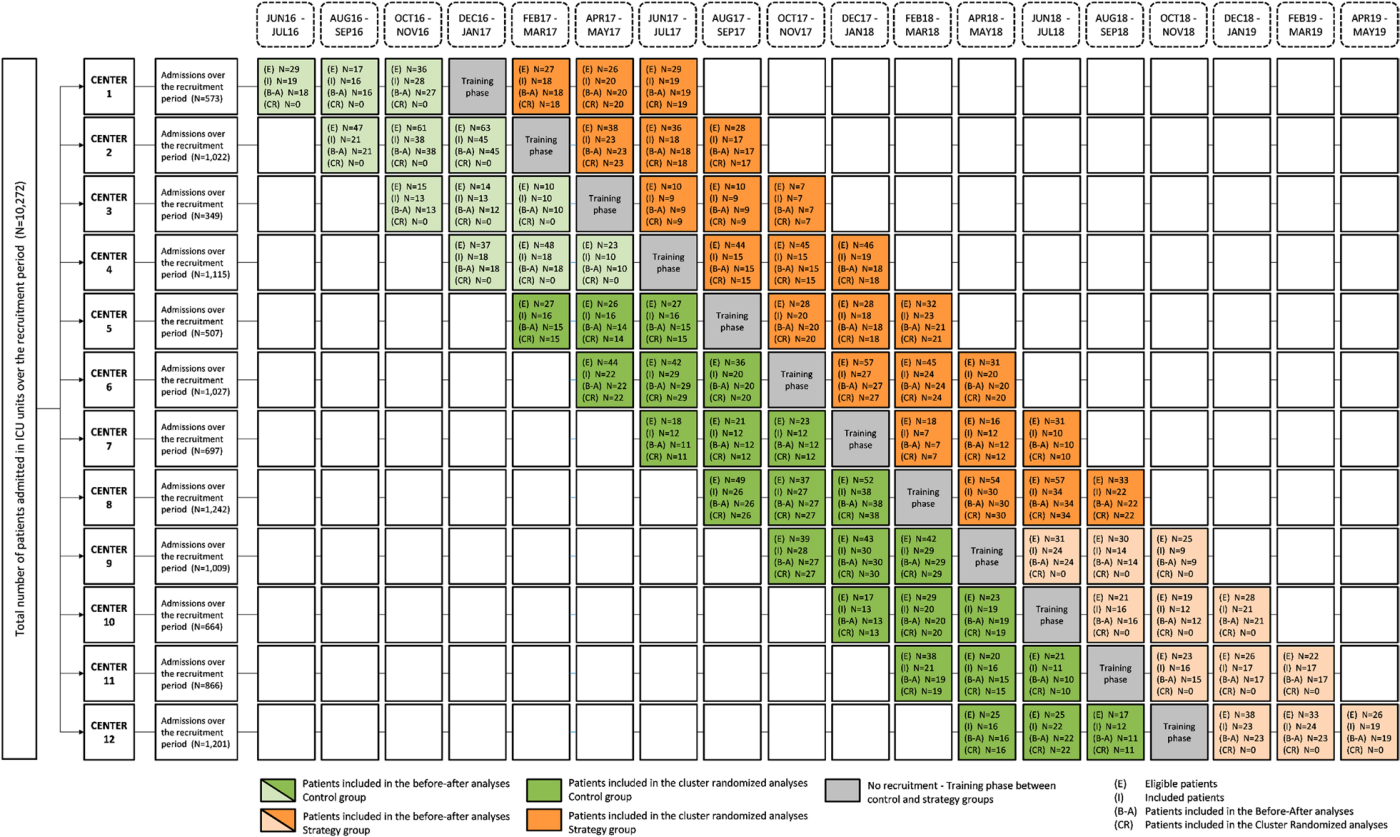
CONSORT diagram of patient recruitment in the POINCARE-2 trial

For feasibility purposes, the recruitment period was restricted to 1 year in each ICU. Relying on a previously published design [20], we handled the period effect pertaining to the stepped wedge design by two methods. First, by comparing the same cluster-periods in strategy versus control groups (main analyses, further called cluster-randomized analyses [CRA]). Second, by taking into account the period effect using mixed models in the whole sample, thus mimicking a quasi-experimental before-and-after analysis that would have been randomized in a stepped wedge fashion (secondary analyses, further referred as randomized before-and-after analyses [RBAA], Fig. 1).

We presented the trial design in Fig. 1. The cluster was the ICU. We considered 12 sequences, and one ICU was randomized at each sequence. Each ICU contributed to the recruitment of patients during six cluster-periods.

### Patients

Eligible ICUs were French ICUs that volunteered to participate in the trial.

Inclusion criteria for patients were: age ≥ 18 years old, under mechanical ventilation (through endotracheal tube), admission to one of the 12 recruiting ICUs for > 48 and ≤ 72 h, and expected length of stay after inclusion > 24 h. Exclusion criteria were clinical condition or unavailability of bedside scale impeding weighing, multiple trauma, hospital stay in another ICU > 24 h immediately preceding the index ICU admission, pregnancy, expected withdrawal of life-sustaining therapy within 7 days after admission, patient refusal of personal data collection (or use), history of ICU stay in one of the 12 recruiting ICUs during the recruitment period, and under guardianship.

One of the investigators of the concerned ICU informed eligible patients and their kin about the trial protocol and their right to refuse to participate in the trial. Because the POINCARE-2 strategy focused on health care organization, written informed consent was waived in accordance with the French law (Bill number 2012–300 on March 5, 2012 about research involving humans).

### Randomization

Assignment of the POINCARE-2 strategy was at the cluster level and performed by JMV. PEB recruited volunteer ICUs. JMV used computer-generated numbers to randomly assign each ICU to one of the 12 sequences. ICU staff were informed about their own allocation 1 month before their time of inclusion. ICUs were blinded to other ICUs allocation (until allocation occurrence). Once an ICU was assigned to a sequence, the research team met with the whole ICU staff and the dedicated onsite research nurse to inform them about the trial procedures for the control period during the month before the time of inclusion. The control period started at this time at a cluster level. The control period was followed by a training period (Additional file 1: Fig. S1) during which the research team planned a second meeting with the ICU staff to deliver information and complete documentation (posters displayed in ICU rooms, and brochures) about the strategy to implement (Additional file 1: Fig. S1) to enhance adherence to the strategy. We delivered full information about the rationale and ways to deliver the strategy to all the patients admitted to the ICU. The strategy period started at the end of the training period at a cluster level. Once the strategy was implemented in a participating ICU, all patients admitted to this ICU were supposed to receive the POINCARE-2 strategy.

Investigators continuously recruited participants over 6 periods (i.e., 3 cluster-periods contributing to the control group, then 3 cluster-periods contributing to the strategy group). All participants recruited over one cluster-period were included in either the control or the strategy group, depending on date of admission to the recruiting ICU (Fig. 1).

### Procedures

The POINCARE-2 strategy is summarized in Additional file 1: Fig. S1 [19]. It was delivered at a patient level. It relied on daily weighing from Day 2 to Day 14 after admission and subsequent daily decision to restrict salt and water and to administer diuretics (i.e., furosemide 1 mg/kg) and/or albumin (i.e., albumin 20% 100 mL/8 h for 24 h at maximum) in case of excessive weight gain. Body weight gain was considered a marker of fluid retention. Daily body weight was assessed by trained nurses using intensive care beds with integrated weighing scales, when available; ceiling hoist scales; or bed scales. Water and salt restriction, diuretics, and albumin were prescribed by an ICU senior intensivist MD or by ICU residents supervised by an ICU senior intensivist MD. Water and salt restriction pertained to intravenous fluid and included fluid bolus administration, maintenance fluid, and carrier fluid for medications. For safety purposes, withholding the strategy until recovery was recommended in case of: arterial hypotension, defined as a systolic arterial pressure < 90 mmHg and/or vasopressor use at the discretion of the intensivist, serum potassium level < 2.8 mmol/L, serum sodium level > 155 mmol/L, incident deterioration of renal function with an “injury” level of the RIFLE classification, or any adverse event (at the discretion of the intensivist).

Patients of the control group received standard care, as routinely delivered by each ICU staff in usual practice.

### Data collection

Baseline patient characteristics collected consisted of age, sex, weight (kg), Mc Cabe score (A, B, C), coexisting conditions, Simplified Acute Physiology Score (SAPS) II, Sepsis-related Organ Failure Assessment (SOFA), and main cause of admission to the ICU. Serum sodium level (mmol/L), serum potassium level (mmol/L), serum bicarbonate level (mmol/L), serum creatinine level (mg/dL), and arterial PaO_2_:FiO_2_ (mmHg) were collected daily, and their worst value was considered in case of multiple assessment per day. Treatment with vasopressors, renal replacement therapy and mechanical ventilation were also collected daily until Day 60.

### Adherence to the POINCARE-2 strategy

Independent research nurses collected daily body weight, biological assays, and treatment prescriptions from Day 0 to Day 14 from medical records. To assess contamination during the control period (i.e., whether POINCARE-2 strategy was already partially implemented before the planned implementation date), data concerning components of POINCARE-2 strategy (i.e., body weight, biological assays, and treatment prescriptions) were also collected by independent research nurses for patients included during the control period. Weight was routinely collected daily from Day 0 to Day 14 in the strategy group, and on Day 0, Day 7, and Day 14 in the control group (or more frequently when weighing was performed more frequently as part of routine care during the control period).

To assess adherence to the POINCARE-2 strategy once the strategy was implemented, we used fluid balance estimates and furosemide and albumin prescription as indicators of adherence. Fluid balance was assessed by using (i) weight evolution between Day 0 and Day 7, defined as the difference between weight at Day 7 and weight at Day 0; (ii) weight evolution between Day 0 and Day 14, defined as the difference between weight at Day 14 and weight at Day 0; (iii) average fluid balance from Day 0 to Day 7, defined as individual arithmetic mean of daily fluid balance (i.e., difference between individual volume of fluid intake and output) over 7 days after Day 0; and (iv) average fluid balance from Day 0 to Day 14, defined as individual arithmetic mean of daily fluid balance (i.e., difference between individual volume of fluid intake and output) over 14 days after Day 0. Furosemide and albumin prescriptions were assessed using (i) total dose of diuretics from Day 0 to Day 14, defined as the cumulated dose of diuretics administered to a patient over 14 days after Day 0, and (ii) total dose of albumin 20% from Day 0 to Day 14, defined as the cumulated dose of albumin 20% administered to a patient over 14 days after Day 0.

### Follow-up and outcomes

The primary outcome was 60-day all-cause mortality. Vital status and date of event in case of death were extracted from medical records or collected by investigators by phone calls to patients or their family.

Secondary outcomes were 28-day and in-hospital all-cause mortality; MVFDs, defined as the cumulative number of days alive with no mechanical ventilation between Day 0 and Day 28; vasopressor-free days (VFDs), defined as the cumulative number of days alive with no prescription between Day 0 and Day 28; and renal replacement therapy-free days (RRTFDs), defined as the cumulative number of days alive with no renal replacement therapy between Day 0 and Day 60. Patients dying within the assessment intervals, and who were under mechanical ventilation, vasopressor prescription, or renal replacement therapy at the time of death, were assigned a count of zero free days, respectively, for the variable of interest.

Safety outcomes consisted of unexpected harmful events occurring at least once between Day 2 and Day 14: arterial hypotension, defined as arterial systolic pressure < 90 mmHg; hypernatremia, defined as serum sodium level > 155 mmol/L; hypokalemia, defined as serum potassium level < 2.8 mmol/L; renal damage, defined by a worsening in the RIFLE criteria during Day 3–Day 14 compared to the higher RIFLE criteria during Day 1 and Day 2 [21]; and acute ischemic events (defined as myocardial infarction and/or patent mesenteric ischemia).

### Statistical analysis

We split analyses between CRA (main analyses) and RBAA (secondary analyses). All analyses were intention-to-treat.

Baseline characteristics and adherence to the strategy were described in each group (strategy vs. control), and compared by using the absolute value of the standardized difference (Stdiff), with a Stdiff > 0.1 considered as a marker of imbalance between groups. We described categorical variables with frequencies and percentages, and continuous variables with mean and standard deviation (SD) in case of normal distribution, or median and interquartile range (IQR) otherwise.

We described outcomes in each group (strategy vs. control) with frequencies and percentages for categorical variables, median and IQR for continuous variables, or Kaplan Meier estimates for survival.

To assess the effect of the strategy, we used log-binomial mixed models (or modified Poisson regressions in case of non-convergence) for binary outcomes, and zero-inflated negative binomial (or zero-inflated Poisson) mixed models for continuous outcomes. We relied on two-level crude models, with ICU (and 2-month period effect in RBAA only) entered as a random effect and intervention group as a fixed effect. As planned, we entered additional unbalanced baseline characteristics (age, SAPS II, and McCabe score) as fixed effects in adjusted models. We presented the results as exp(parameters) with their 95% confidence intervals (CIs). For binary outcomes analyzed with log-binomial or modified Poisson mixed models, exp(parameters) can be interpreted as risk-ratios [RRs]. For continuous outcomes analyzed with zero-inflated models, exp(parameters) can be interpreted as odds ratios (OR) for the logistic zero-excess part of the model and as a multiplicative factor of the counting process for the negative binomial part of the model.

We imputed missing outcomes based on the maximal bias hypothesis. In multivariable analyses, we treated missing unbalanced baseline characteristics by multiple imputation. *p*-values were two-sided, and statistical significance was set at 0.05 for the primary outcome in the main analyses, and for safety outcomes. In all other analyses, we used Bonferroni adjustment. We performed all analyses using SAS© 9.4 (SAS Institute, Inc, Cary, NC, USA).

Assuming an expected 60-day mortality of 37.5% [22–24] in the control group, a coefficient of variation *k* of 0.26, alpha set at 0.05, a power set at 0.8, and a minimal 15% absolute reduction [1, 3, 4, 23] in mortality expected in the strategy group, a total of 917 participants recruited over 48 cluster-periods was required for CRA.

We monitored data for all included patients while the trial was ongoing (list of queries available upon request).

The POINCARE-2 trial was registered at ClinicalTrials.gov (NCT 02,765,009).

## Results

**Fig. 2:**
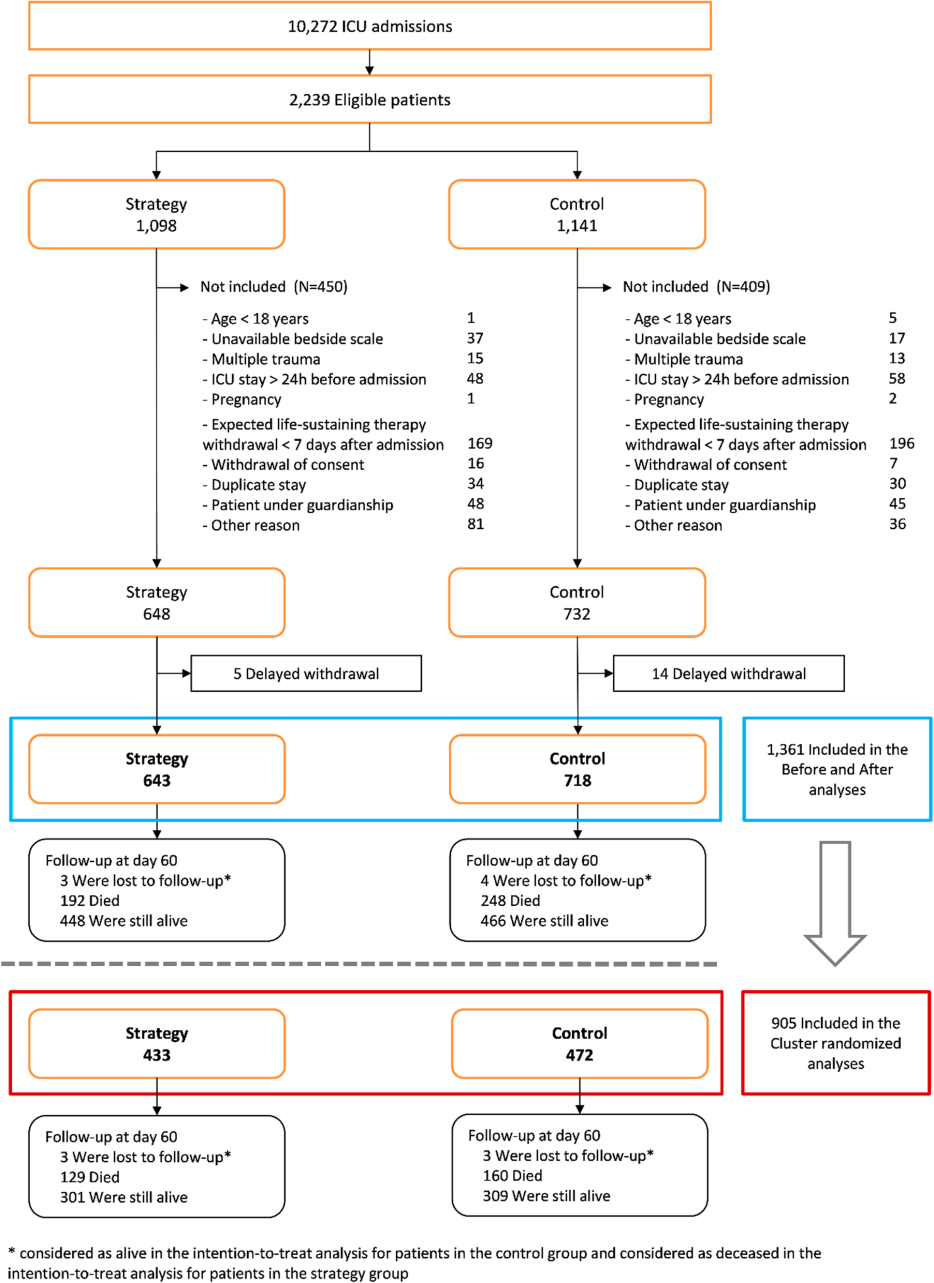
Flowchart of critically ill patients recruited and followed up in the POINCARE-2 trial

A total of 10,272 patients were admitted to one of the 12 recruiting ICUs over the recruitment period held from June 1, 2016, to May 31, 2019 (Fig. 2); 1361 were included in the RBAA (643 in the strategy group and 718 in the control group) and 905 in the CRA (433 in the strategy group and 472 in the control group). Recruitment is detailed by cluster-period in Fig. 1.

Baseline characteristics of patients included in the CRA are described in Table 1 (missing data reported in Additional file 1: Table S2). As compared with the control group, the strategy group was younger and had lower SAPS II and poorer McCabe score. They also had lower potassium level and higher bicarbonates level. In addition, they were more often admitted to the ICU due to central nervous system (CNS) injury than to any other causes. RBBA led to similar results, except for age and SAPS II, which showed a StDiff < 0.1 (see Additional file 1: Tables S1 and S2).

**Table 1:** Baseline characteristics of patients included in cluster-randomized analyses of the POINCARE-2 trial

	Control group, No. (%) (*n* = 472)	Strategy group, No. (%) (*n* = 433)	Stdiff
Age, mean (SD), y	65.7 (14.3)	63.7 (14.1)	0.15
Male sex	308 (65.3)	278 (64.2)	0.02
Weight at ICU admission, mean (SD), kg	79.4 (23.5)	78.5 (20.0)	0.04
Height, mean (SD), cm	169.8 (9.5)	170.1 (9.4)	0.03
Mc Cabe score^a^			0.20
Unknown	32	31	
A	263/440 (59.8)	279/402 (69.4)	
B	143/440 (32.5)	98/402 (24.4)	
C	34/440 (7.7)	25/402 (6.2)	
Coexisting conditions			
Congestive heart failure	38/452 (8.4)	37/419 (8.8)	0.02
Chronic respiratory failure	92/463 (19.0)	80/423 (18.9)	0.02
Chronic kidney disease	12/466 (2.6)	8/425 (1.9)	0.05
Diabetes mellitus	131/464 (28.2)	102/425 (24.0)	0.10
Cirrhosis	32/457 (7.0)	26/419 (6.2)	0.03
Immunodeficiency	64/463 (13.8)	63/426 (14.8)	0.03
Cancer	78/462 (16.9)	78/422 (18.5)	0.04
SAPS II at day 1, median (IQR)^b^	60.0 (47.0–72.0)	56.0 (45.0–69.0)	0.15
SOFA score at admission^c^, median (IQR)	9.0 (6.0–12.0)	9.0 (6.0–11.0)	0.08
Serum sodium level, mean (SD), mmol/L	137.8 (6.7)	138.1 (6.6)	0.03
Serum potassium level, mean (SD), mmol/L	4.2 (1.0)	4.0 (0.9)	0.22
Serum bicarbonate level, mean (SD), mmol/L	20.8 (7.4)	22.0 (6.4)	0.17
Serum creatinine level, median (IQR), mg/dL	1.1 (0.7–1.9)	1.0 (0.7–1.7)	0.10
PaO_2_:FiO_2_, mean (SD), mmHg	216.9 (140.8)	214.5 (133.3)	0.02
Main cause of admission			0.23
Septic shock^d^	88 (18.6)	70 (16.2)	
ARDS or acute respiratory failure	172 (36.4)	151 (34.9)	
Heart failure	36 (7.6)	22 (5.1)	
Acute renal failure	10 (2.1)	8 (1.8)	
Post-surgery	14 (3.0)	20 (4.6)	
CNS injury	118 (25.0)	139 (32.1)	
Other	34 (7.2)	23 (5.3)	
Vasopressors at day 0	268 (56.8)	236 (54.5)	0.05
Renal replacement therapy at day 0	33 (7.0)	25 (5.8)	0.05
ICU length of stay, median (IQR), days	12 (8–20)	11 (7–21)	

Stdiff denotes standardized difference (absolute value)

*ARDS* Acute respiratory distress syndrome, *CNS* Central nervous system, *ICU* Intensive care unit, *IQR* Interquartile range

^a^McCabe score of A indicates no underlying disease that compromises life expectancy, B an estimated life expectancy with the chronic disease of less than 5 years, and C an estimated life expectancy with the chronic disease of less than 1 year. ^b^Simplified Acute Physiology Score (SAPS) II ranges from 0 to 164, with higher scores indicating greater severity of symptoms. ^c^Sepsis-related Organ Failure Assessment (SOFA) score ranges from 0 to 24, with higher scores indicating more severe organ failure. ^d^Septic shock was defined as sepsis-related hypotension despite emergency fluid loading, requiring a vasopressor

**Fig. 3:**
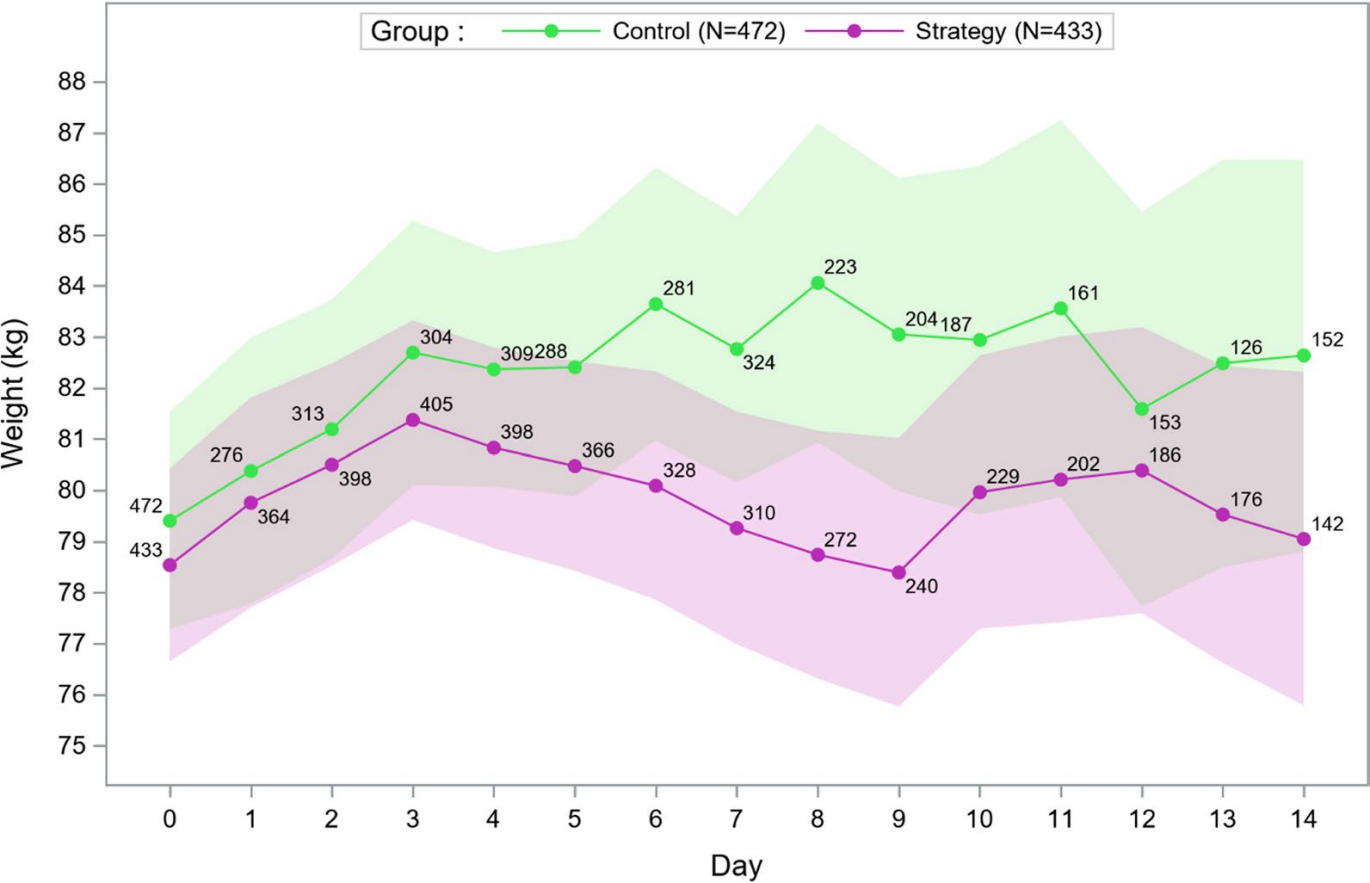
Evolution of the average weight from Day 0 to Day 14 in patients included in cluster-randomized analyses of the POINCARE-2 trial (with 95% confidence interval of mean and sample size as point label). Using a mixed model entering the ICU and patient as random effects and the time period (defined as ≤ 2 vs. > 2 days) interaction with the linear time as a fixed effect, we found no difference in trend between the 2 groups during the second time period (> 2 days): *p* = 0.39

Adherence to the POINCARE-2 strategy is detailed in Additional file 1: Table S3. In CRA, mean ± SD weight difference between Day 7 and Day 0 was 1.2 ± 6.1 kg in the strategy group versus 2.3 ± 7.2 kg in the control group (mean difference − 1.1, 95% CI − 2.7 to 0.5, *p* = 0.70) (Additional file 1: Table S3). We observed a trend to lower mean daily weight in the strategy than in the control group, which was not statistically significant (Fig. 3 for CRA, Additional file 1: Fig. S2 for RBAA). In CRA, the mean difference in average daily fluid balance between groups was higher from Day 0 to Day 7 (− 183 mL, 95% CI − 368 to 26) than from Day 0 to Day 14 (− 64 mL, 95%CI − 226 to 97). RBBA led to similar results (− 242 mL, 95% CI − 392 to − 92 and − 164 mL, 95% CI − 294 to − 33, respectively). Observed cumulative fluid balance from Day 0 to Day 14 was lower but not significantly in the strategy than control group (Additional file 1: Fig. S3 for CRA, and Fig. S4 for RBAA).

In CRA, from Day 0 to Day 14, a median total dose of furosemide of 220 mg (IQR 80 to 520) was administered to 314 patients in the strategy group versus 305 mg (IQR 80–727) to 328 patients in the control group. Over the same period, a median of total infused 20% albumin of 550 mL (IQR 300–1000) was administered to 60 patients in the strategy group versus 400 mL (IQR 300–1000) to 47 patients in the control group. Similar results were observed in RBAA, except for furosemide, which was no longer less prescribed in the strategy than control group [233 mg (IQR 80–590) to 478 patients vs. 220 mg (IQR 80–620) to 475 patients, respectively, Additional file 1: Table S3].

Results for the primary, secondary, and safety outcomes of CRA (RBAA) are summarized in Table 2 (Additional file 1: Table S4).

**Table 2:** Primary, Secondary, and Safety Outcomes in cluster randomized analyses of the POINCARE-2 trial

	Control group % [95% CI]^b^	Strategy group % [95% CI]^b^	Crude effect of strategy					Adjusted effect of strategy^a^				
	Median (IQR)^c^	Median (IQR)^c^	Model (part)		Exp(parameter) [95% CI]		*p*-value	Model (part)		Exp(parameter) [95% CI]		*p*-value
*Primary outcome*												
60-day mortality rate^b^	33.9 [29.6–38.2]	30.5 [26.2–34.8]	MP		RR	0.90 [0.75–1.08]	0.26	MP		RR	0.90 [0.75–1.08]	0.99
*Secondary outcomes*												
28-day mortality rate^b^	26.7 [22.7–30.7]	21.7 [17.8–25.6]	MP		RR	0.77 [0.56–1.06]^d^	0.26^d^	MP		RR	0.77 [0.56–1.06]^d^	> 0.99^d^
In-hospital mortality rate^b^	32.6 [28.4–36.9]	29.6 [25.3–33.9]	MP		RR	0.91 [0.68–1.21]^d^	> 0.99^d^	MP		RR	0.91 [0.68–1.21]^d^	> 0.99^d^
Mechanical ventilator-free days^c^	9.0 [0.0–18.0]	9.0 [0.0–17.0]	ZINB	(NB)	MF	1.01 [0.91–1.12]^d^	> 0.99^d^	ZINB	(NB)	MF	1.01 [0.91–1.12]^d^	> 0.99^d^
				(ZI)	OR	0.91 [0.57–1.46]^d^	> 0.99^d^		(ZI)	OR	0.91 [0.57–1.46]^d^	> 0.99^d^
Vasopressor-free days^c^	17.0 [7.0–24.0]	17.0 [8.0–26.0]	ZINB	(NB)	MF	1.03 [0.96–1.10]^d^	> 0.99^d^	ZINB	(NB)	MF	1.03 [0.96–1.10]^d^	> 0.99^d^
				(ZI)	OR	0.84 [0.42–1.68]^d^	> 0.99^d^		(ZI)	OR	0.84 [0.42–1.68]^d^	> 0.99^d^
Renal replacement therapy-free days^c^	23.0 [11.0–47.5]	23.0 [11.0–49.0]	ZIPoi	(Poi)	MF	0.996 [0.97–1.03]^d^	> 0.99^d^	ZIPoi	(Poi)	MF	0.996 [0.97–1.03]^d^	> 0.99^d^
				(ZI)	OR	1.11 [0.40–3.11]^d^	> 0.99^d^		(ZI)	OR	1.11 [0.40–3.11]^d^	> 0.99^d^
*Safety outcomes*												
Arterial hypotension^b^	77.8 [74.0–81.5]	71.6 [67.4–75.9]	MP		RR	0.94 [0.85–1.03]	0.16	MP		RR	0.94 [0.85–1.03]	0.39
At least one episode of hypernatremia> 155 mmol/l^b^	2.3 [1.0–3.7]	5.8 [3.6–8.0]	LB		RR	2.72 [1.23–6.00]	0.01	LB		RR	2.72 [1.23–6.00]	0.02
At least one episode of hypokalemia < 2.8 mmol/l^b^	7.6 [5.2–10.0]	7.2 [4.8–9.6]	LB		RR	1.02 [0.61–1.72]	0.93	LB		RR	1.02 [0.61–1.72]	0.60
Renal damage ^b,e^	20.3 [16.6–24.0]	23.5 [19.5–27.6]	LB		RR	1.16 [0.90–1.50]	0.25	LB		RR	1.16 [0.90–1.50]	0.24
Myocardial infarction^b^	0.4 [0.0–1.0]	1.15 [0.2–2.2]	LB		RR	1.82 [0.23–14.15]	0.57	LB		RR	1.82 [0.23–14.15]	0.61
Mesenteric ischemia^b^	0.6 [0.0–1.4]	0.2 [0.0–0.7]	LB		RR	0.36 [0.04–3.49]	0.38	LB		RR	0.36 [0.04–3.49]	0.45

*CI* Confidence interval, *IQR* Interquartile range, *MP* Mixed modified Poisson, *RR* Risk ratio, *ZINB* Zero-inflated negative binomial mixed, *NB* Negative binomial part, *MF* Multiplicative factor, *ZI* Zero-inflated part, *ZIPoi* Zero-inflated Poisson mixed, *Poi* Poisson part, *LB* Log-binomial mixed

^a^Adjusted for the following baseline characteristics: age, main cause of admission (recoded as CNS injury vs. other causes of admission), McCabe score, SAPS II at day 1

^b^Binary outcomes: log-binomial (modified Poisson) mixed models were used and “exp(parameter)” are estimations of RR

^c^Numeric outcomes: modeled with zero-inflated negative binomial (Poisson) mixed models which combine a logistic regression and a negative binomial (Poisson respectively) model and “exp(parameter)” are odds ratios of excess of zeros for the zero-inflated part (logistic) and multiplicative factor for the counting part (negative binomial/Poisson)

^d^Bonferroni correction was used: Confidence intervals according to *α* = 5/22 = 0.23% and *p*-values by a multiplying a coefficient of 22

^e^Renal damage was defined by worsening RIFLE between Day 3 and Day 14 as compared with the higher RIFLE observed on Day 1 or Day 2. For renal damage, analyses were conducted within the subsample of at-risk patients (i.e., in 883/905 patients not classified as RIFLE “End Stage Kidney Disease” at Day 2)

A total of 129 patients died in the strategy group (vs. 160 in the control group) during the first 60 days of follow-up. Three patients were lost to follow-up in the strategy group and further considered dead (vs. four patients in the control group, further considered alive). Survival estimates in the 2 groups are displayed in Additional file 1: Fig. S5 for CRA (and Additional file 1: Fig. S6 for RBAA). In CRA, 60-day mortality rates were 30.5% (95% CI 26.2–34.8) for the strategy group versus 33.9% (95% CI 29.6–38.2) for the control group (RR = 0.90, 95% CI, 0.75–1.08, *p* = 0.26, Table 2). Adjustment for unbalanced baseline characteristics did not modify this result (RR = 1.00, 95% CI 0.86–1.17, *p* = 0.99, Table 2). RBAA led to similar results (Additional file 1: Table S4).

In CRA, 28-day and in-hospital mortality, MVFDs, VFDs, and RRTFDs did not differ between groups (Table 2). RBAA led to similar results (Additional file 1: Table S4).

In CRA, the occurrence of arterial hypotension, hypokalemia, renal damage, and myocardial or mesenteric infarction did not differ between groups (Table 2). However, hypernatremia was more frequent in the strategy than control group (RR = 2.72, 95% CI 1.23–6.00, *p* = 0.01). Adjustment for potential confounders did not modify this result (Table 2). RBAA led to similar results (Additional file 1: Table S4).

## Discussion

Intention-to-treat CRA and RBAA led to insignificant results regarding the effect of the POINCARE-2 strategy on 60-day mortality and all secondary outcomes, except hypernatremia.

To our knowledge, POINCARE-2 is the largest trial assessing the effectiveness of a strategy targeting fluid balance control in a broad range of critically ill patients. A recent meta-analysis by Silversides et al. [25] reported that conservative strategies (vs. liberal strategies) had no effect on mortality. However, conservative strategies were associated with increased MVFDs and reduced ICU length of stay. Despite evidence of compliance with the strategy (i.e., lower mean weight and lower fluid balance in the strategy than control group), we did not find any statistical difference between groups regarding mortality or secondary effectiveness outcomes. This observation could be explained by actual ineffectiveness of the strategy under scrutiny, or actual effectiveness of the strategy combined with an inability to prove it using intention-to-treat analyses.

Intention-to-treat analyses are commonly used to assess effectiveness of interventions. Considering patients from the control group unexposed to the strategy and patients from the strategy group optimally exposed, whatever their actual exposure to the strategy, can result in attenuated between-group differences [26]. Accordingly, positive significant results of randomized clinical trials can be transferred to clinical practice with low residual doubt about actual efficacy of the strategy. However, insignificant results of such analyses do not allow for firm conclusions about the actual effect of the strategy, especially in case of switching between intervention groups [26], which can result in incomplete separation between groups. This might explain (i) the statistically insignificant results of POINCARE-2 trial, and (ii) why the POINCARE-2 strategy should not be discarded yet.

First, although less frequent, daily weighing was also performed during the control period; and both furosemide and albumin were also prescribed during the control period as part of standard of care, resulting in possible contamination (i.e. exposure to [at least part of] the POINCARE-2 strategy during the control period). In addition, CRA results showed that furosemide was prescribed more in the control than strategy group, which could result from a greater need of diuretics in patients without water/salt restriction, or by residual unbalance between groups. Because furosemide was slightly (though not significantly) more prescribed in the strategy group in RBAA (Additional file 1: Table S3), and the intra-period correlation coefficient was lower than the intra-cluster correlation coefficient (Additional file 1: Table S5), the latter explanation is more likely. However, the lower fluid intake observed in the strategy group (Additional file 1: Table S3) is probably related to a fair compliance with fluid restriction included in the strategy protocol in case of weight gain and likely explained the lower need of diuretics in the strategy group. Second, we observed better compliance with the protocol during the first week than during the second week in the strategy group, thus resulting in suboptimal exposure to the strategy. Third, observed mortality estimates were lower in the strategy group than in the control group (30.5% vs. 33.9%), as were 28-day mortality estimates (21.7% vs. 26.7%), which argues for a non-null clinical effect of the strategy.

Therefore, contamination during the control period and suboptimal application of the POINCARE-2 strategy during the strategy period might have resulted in incomplete group separation and subsequent underestimation of the POINCARE-2 strategy effect. In addition, the POINCARE-2 strategy was flexible and its application was left at the discretion of the intensivist to meet the patients’ needs. Accordingly, during the strategy period, departure from the strategy protocol might have occurred in patients not ready for de-resuscitation on Day 2, such as patients with severe septic shock, thus resulting in apparent suboptimal application of the strategy and further incomplete separation between groups. At this point, only additional results from per-protocol, as-treated, and subgroup analyses, as planned in the POINCARE-2 protocol [19], could help further discuss the actual effectiveness of the POINCARE-2 strategy.

To avoid a too early “de-resuscitation”, the POINCARE-2 strategy was started at Day 2 after ICU admission. Both mean (Day 7–Day 0) weight difference and average fluid balance were positive but lower than expected, as compared to that observed in trials included in the meta-analysis by Silversides et al [25]. Five of these trials reported a net between-group fluid balance difference of < 1500 mL, 4 trials a difference between 1500 and 4000 mL, and 2 trials a difference reaching 7000 mL. Of note, there was no clear association between the intensity of water restriction and outcomes. A trial resulting in a 7000 mL difference between groups [2] showed a statistically insignificant 3% difference in 60-day mortality favoring the conservative strategy. The effect of conservative strategies on mortality might follow a J-shape curve, like that observed for BMI or blood pressure effect, with a higher risk of mortality in strategies that are either too restrictive, or too liberal regarding fluid balance control.

Although the timing for fluid management was strict in the POINCARE-2 strategy, the strategy itself was more of a set of recommendations than a restrictive standardized procedure. Although hypothetical, intensivists’ attitudes toward the POINCARE-2 strategy could explain part of the observed contamination: in other words, they might have been convinced of the beneficial effect of a conservative approach before the POINCARE-2 trial started, and might have implemented it earlier than planned during the trial. Only a complete process evaluation [27] could help untangle contextual factors (e.g., institutional policies, ICU environment influencing weighing, or intensivists’ attitudes toward fluid control in critically ill patients) that might have influenced adherence to POINCARE-2 strategy.

Potential deleterious consequences of conservative fluid management or de-resuscitation include hemodynamic disturbances, deterioration of renal function, and electrolyte disturbances. The occurrence of arterial hypotension, major ischemic events, and renal damage, as well as VFDs and RRTFDs, did not differ between groups, in both CRA and RBAA. Altogether, these results suggest that a conservative approach was safe for hemodynamic and renal function.

Severe hypernatremia was more frequent in the strategy group whatever the analyses. Similar results were observed in the trial conducted by the ARDS network [2], and a recent pilot study of protocoled diuresis for de-resuscitation in ICU [28]. Increasing evidence suggests that acquired-hypernatremia may increase mortality [29] as soon as serum sodium level exceeds 145 mmol/L. The mechanisms underpinning the deleterious effects of hypernatremia remain unclear, but hypernatremia correction is independently associated with better survival [30].

The POINCARE-2 trial has some strengths. First, the number of critically ill patients were prospectively included in a timely manner, as planned, which is critical in a stepped wedge trial and could otherwise compromise the design. Second, the thorough monitoring of the data by an ISO 9001-certified research lab (*CIC, Epidémiologie clinique*) ensured their accuracy. Third, additional process evaluation, as-treated, and subgroup analyses were planned a priori, and will help further discuss the implementation, underpinning mechanisms of the strategy, context of implementation, and actual effectiveness of the POINCARE-2 strategy with regard to the results presented in this article.

However, the POINCARE-2 trial also has some limitations.

First, the trial was powered to detect a 15% absolute difference in 60-day mortality, which might seem high with regard to the 3% difference actually observed.

The magnitude of the difference that was assumed to determine the size of the POINCARE-2 trial was based on results of previous studies assessing fluid balance control [1] that were available at the time of the study conception, but may have led to an overoptimistic estimation of the required sample size. The lack of significance in our results could thus be related to a lack of statistical power induced by this estimation.

Second, the intra-period correlation coefficient was lower than the intra-cluster correlation coefficient. This situation might threaten the validity of CRA. These analyses were chosen as primary analyses in 2013, at the time the trial got funded, on the basis of a previous publication [20]. However, results from RBAA were consistent with CRA results, which lowers the impact of this eventual threat.

Third, contamination might have threatened the validity of the intention-to-treat analyses. Although assessment of adherence to the strategy suggested some difference in fluid accumulation between groups, these differences were statistically non-significant, resulting in a lower than anticipated difference in strategy effect between groups. One could argue that a better monitoring of adherence to the strategy would have prevented such contamination. However, adherence of investigators and ICU nurses to conservative strategies might have affected their will to participate in the trial and their implementation of the strategy. In addition, components of the POINCARE-2 strategy are commonly used in ICUs. Unfortunately, even if stepped wedge randomized controlled trials are best fit to handle investigators’ preference toward the intervention under scrutiny, they cannot totally prevent its anticipated implementation.

Fourth, the POINCARE-2 strategy was based on a pragmatic approach, targeting fluid balance control by using few human and material resources. Accordingly, daily weighing was used as a proxy of fluid balance assessment. Although cumulative fluid balance evolution from Day 0 to Day 14 corroborated findings from average weight evolution over the same period in our results, we cannot firmly state that weight was an accurate proxy of fluid balance in ICU patients.

## Conclusion

Relying on intention-to-treat analyses, the POINCARE-2 conservative strategy did not reduce mortality in critically ill patients. However, an overly optimistic estimate of the expected difference in effect, as well as a smaller than expected difference in the strategy delivered between the two groups, could bias our results toward the null, and explain their non-significance. Besides, intention-to-treat analyses might not reflect the actual exposure to the strategy. As-treated analyses might help address contamination or lack of adherence of health professionals to the strategy and could strengthen the conclusions regarding what is best for critically ill patients.

## Supplementary Information

**Additional file 1: Fig. S1.** POINCARE-2 strategy of fluid balance control in critically ill patients (reproduced from 19). **Table S1.** Baseline characteristics of patients in randomized before-and-after analyses of the POINCARE-2 trial. **Table S2.** Description of missing data at baseline for patients in cluster-randomized (CRA) and randomized before-and-after (RBAA) analyses of the POINCARE-2 trial. **Table S3.** Adherence to the POINCARE-2 strategy in cluster-randomized and randomized before-and-after analyses. **Fig. S2.** Evolution of the average weight from Day 0 to Day 14 in patients in randomized before-and-after analyses of the POINCARE-2 trial (with 95% confidence interval of mean and sample size as point label). **Fig. S3.** Smoothed cumulated fluid balance evolution from Day 0 to Day 14 in patients in cluster-randomized analyses of the POINCARE-2 trial (with 95% confidence interval of mean prediction). **Table S4.** Primary, Secondary, and Safety Outcomes in randomized before-and-after analyses of the POINCARE-2 trial. **Table S5.** Intra-cluster, intra-period and intra-cluster-period correlation for 60-day mortality. **Fig. S4.** Smoothed cumulated fluid balance evolution from Day 0 to Day 14 in patients in randomized before-and-after analyses of the POINCARE-2 trial (with 95% confidence interval of mean prediction). **Fig. S5.** Survival of patients included in cluster-randomized analyses of the POINCARE-2 trial. **Fig. S6.** Survival of patients included in randomized before-and-after analyses of the POINCARE-2 trial.

## Data Availability

Due to restrictions pertaining to French laws, the datasets generated and/or analyzed during POINCARE-2 trial are not publicly available. However, data transfer agreement remains possible, and data can be made available upon reasonable request to the corresponding author.

## Abbreviations

ARDS: Acute respiratory distress syndrome
CRA: Cluster-randomized analyses
ICU: Intensive care unit
MVFD: Mechanical ventilator-free days
RBAA: Randomized before-and-after analyses
RRTFD: Renal replacement therapy-free day
VFD: Vasopressor-free day
