# Supplementary material for: Fluid balance control in critically ill patients: results from POINCARE-2 stepped wedge cluster-randomized trial
Source: Crit Care. 2023 Feb 21;27:66. doi: 10.1186/s13054-023-04357-1 (PMC9945675; doi:10.1186/s13054-023-04357-1)

TABLE OF CONTENTS OF SUPPLEMENTAL ELECTRONIC MATERIAL

[TABLES 3](#_Toc124858188)

[Table S1 – Baseline characteristics of patients in randomised before-and-after analyses of the POINCARE-2 trial 3](#_Toc124858189)

[Table S2 – Description of missing data at baseline for patients in cluster randomised (CRA) and randomised before-and-after (RBAA) analyses of the POINCARE-2 trial 5](#_Toc124858190)

[Table S3 – Adherence to the POINCARE-2 strategy in cluster randomised and randomised before-and-after analyses 6](#_Toc124858191)

[Table S4 – Primary, Secondary, and Safety Outcomes in randomised before-and-after analyses of the POINCARE-2 trial 11](#_Toc124858192)

[Table S5 – Intra-cluster, intra-period and intra-cluster-period correlation for 60-day mortality 14](#_Toc124858193)

[FIGURES 16](#_Toc124858194)

[Figure S1 –POINCARE-2 strategy of fluid balance control in critically ill patients (reproduced from 19) 16](#_Toc124858195)

[Figure S2 – Evolution of the average weight from Day 0 to Day 14 in patients in randomized before-and-after analyses of the POINCARE-2 trial (with 95% confidence interval of mean and sample size as point label) 17](#_Toc124858196)

[Figure S3 – Smoothed cumulated fluid balance evolution from Day 0 to Day 14 in patients in cluster randomised analyses of the POINCARE-2 trial (with 95% confidence interval of mean prediction) 19](#_Toc124858197)

[Figure S4 – Smoothed cumulated fluid balance evolution from Day 0 to Day 14 in patients in randomised before-and-after analyses of the POINCARE-2 trial (with 95% confidence interval of mean prediction) 20](#_Toc124858198)

[Figure S5 – Survival of patients included in cluster randomised analyses of the POINCARE-2 trial 21](#_Toc124858199)

[Figure S6 – Survival of patients included in randomised before-and-after analyses of the POINCARE-2 trial 22](#_Toc124858200)

# TABLES

## Table S1 – Baseline characteristics of patients in randomised before-and-after analyses of the POINCARE-2 trial

|  | Control group, No. (%)  (n=718) | Strategy group, No. (%)  (n=643) | Stdiff |
| --- | --- | --- | --- |
| Age, mean (SD), y | 64.5 (14.8) | 64.3±14.3 | 0.01 |
| Male sex | 470 (65.5) | 409 (63.6) | 0.04 |
| Weight at ICU admission, mean (SD), kg | 78.9 (22.6) | 78.8 (20.8) | 0.003 |
| Height, mean (SD), cm | 169.4 (9.7) | 169.5 (9.6) | 0.008 |
| Mc Cabe score^a^  Unknown  A  B  C | 56  419/662 (63.3)  203/662 (30.7)  40/662 (6.0) | 35  428/607 (70.5)  143/607 (23.6)  36/607 (5.9) | 0.20 |
| Coexisting conditions |  |  |  |
| Congestive heart failure | 65/692 (9.4) | 58/618 (9.4) | <0.001 |
| Chronic respiratory failure | 147/706 (20.8) | 144/630 (22.9) | 0.05 |
| Chronic kidney disease | 16/710 (2.3) | 11/635 (1.7) | 0.04 |
| Diabetes mellitus | 191 (27.1) | 164 (25.9) | 0.03 |
| Cirrhosis | 48/699 (6.9) | 46/626 (7.3) | 0.02 |
| Immunodeficiency | 92/706 (13.0) | 78/634 (12.3) | 0.02 |
| Cancer | 103/702 (14.7) | 99/630 (15.7) | 0.03 |
| SAPS II at day1^b^, median (IQR) | 58.0 (46.0-70.0) | 57.0 (45.0-71.0) | 0.03 |
| SOFA score at admission^c^, median (IQR]) | 9.0 (6.0-12.0) | 9.0 (6.0-11.0) | 0.09 |
| Serum sodium level, mean (SD), mmol/L | 137.8 (6.7) | 137.8 (6.7) | 0.006 |
| Serum potassium level, mean (SD), mmol/L | 4.2±0.9 | 4.1±1.0 | 0.11 |
| Serum bicarbonate level, mean (SD), mmol/L | 21.2±7.1 | 22.2±7.1 | 0.15 |
| Serum creatinine level, median (IQR), mg/dL | 1.2 (0.8-1.9) | 1.1 (0.8-1.8) | 0.08 |
| PaO²:FiO², mean (SD), mmHg | 216.4±146.6 | 209.5±130.0 | 0.05 |
| Main cause of admission |  |  | 0.21 |
| Septic shock^d^ | 133 (18.5) | 107 (16.6) |  |
| ARDS or acute respiratory failure | 283 (39.4) | 238 (37.0) |  |
| Heart failure | 60 (8.4) | 33 (5.1) |  |
| Acute renal failure | 15 (2.1) | 13 (2.0) |  |
| Post-surgery | 23 (3.2) | 33 (5.1) |  |
| CNS injury | 161 (22.4) | 179 (27.8) |  |
| Other | 43 (6.0) | 40 (6.2) |  |
| Vasopressors at Day 0 | 395 (55.0) | 357 (55.5) | 0.01 |
| Renal replacement therapy at Day 0 | 51 (7.1) | 36 (5.6) | 0.06 |
| ICU length of stay, median (IQR), days | 12 (7-20) | 11 (7-20) |  |

Notes:

Stdiff denotes standardized difference (absolute value), ARDS acute respiratory distress syndrome, CNS central nervous system, ICU intensive care unit, and IQR interquartile range.

^a^ McCabe score of A indicates no underlying disease that compromises life expectancy, B an estimated life expectancy with the chronic disease of less than 5 years, and C an estimated life expectancy with the chronic disease of less than 1 year

^b^ Simplified Acute Physiology Score (SAPS) II ranges from 0 to 164, with higher scores indicating greater severity of symptoms

^c^ Sepsis-related Organ Failure Assessment (SOFA) score ranges from 0 to 24, with higher scores indicating more severe organ failure

^d^ Septic shock was defined as sepsis-related hypotension despite emergency fluid loading, requiring vasopressor

## Table S2 – Description of missing data at baseline for patients in cluster randomised (CRA) and randomised before-and-after (RBAA) analyses of the POINCARE-2 trial

|  | CRA | | | | RBAA | | | |
| --- | --- | --- | --- | --- | --- | --- | --- | --- |
|  | No. miss |  | % |  | No. miss |  | % |  |
|  |  |  |  |  |  |  |  |  |
| McCabe Score | 63 |  | 7.0 |  | 92 |  | 6.8 |  |
| *Coexisting conditions* |  |  |  |  |  |  |  |  |
| Congestive heart failure | 34 |  | 3.8 |  | 51 |  | 3.7 |  |
| Chronic respiratory failure | 19 |  | 2.1 |  | 25 |  | 1.8 |  |
| Chronic kidney disease | 14 |  | 1.5 |  | 16 |  | 1.2 |  |
| Diabetes mellitus | 16 |  | 1.8 |  | 21 |  | 1.5 |  |
| Cirrhosis | 29 |  | 3.2 |  | 37 |  | 2.7 |  |
| Immunodeficiency | 16 |  | 1.8 |  | 21 |  | 1.5 |  |
| Cancer | 21 |  | 2.3 |  | 29 |  | 2.1 |  |
| SAPS II^a^ | 10 |  | 1.1 |  | 11 |  | 0.8 |  |
| *Biological characteristics* |  |  |  |  |  |  |  |  |
| serum sodium level, mmol/L | 26 |  | 2.9 |  | 36 |  | 2.6 |  |
| Serum potassium level, mmol/L | 29 |  | 3.2 |  | 41 |  | 3.0 |  |
| Serum bicarbonate level, mmol/L | 64 |  | 7.1 |  | 84 |  | 6.2 |  |
| Serum creatinine level, mg/dL | 34 |  | 3.8 |  | 56 |  | 4.1 |  |
| PaO²:FiO², mmHg | 77 |  | 8.5 |  | 101 |  | 7.4 |  |

^a^ SAPS: Simplified Acute Physiology Score

## Table S3 – Adherence to the POINCARE-2 strategy in cluster randomised and randomised before-and-after analyses

|  |  | | | | | | | | | | | | | | | | | | | | | | | | | | | |  | | |  |  |  |
| --- | --- | --- | --- | --- | --- | --- | --- | --- | --- | --- | --- | --- | --- | --- | --- | --- | --- | --- | --- | --- | --- | --- | --- | --- | --- | --- | --- | --- | --- | --- | --- | --- | --- | --- |
|  |  | | | | | | | | | | |  | | | | | | | | | | | | | | |  |  | |  | | | | |
|  |  |  |  |  |  | | |  | | |  | |  | |  | |  | |  | | | |  | | | |  |  | |  | | | | |
|  |  |  |  |  |  |  |  |  |  |  | |  | |  | |  | |  | |  |  |  | |  |  |  |  |  | |  | | | |  |
|  |  |  |  |  |  |  |  |  |  |  | |  | |  | |  | |  | |  |  |  | |  |  |  |  |  | |  | | | |  |
|  |  |  |  |  |  |  |  |  |  |  | |  | |  | |  | |  | |  |  |  | |  |  |  |  |  | |  | | | |  |
|  |  |  |  |  |  |  |  |  |  |  | |  | |  | |  | |  | |  |  |  | |  |  |  |  |  | |  | | | |  |
|  |  |  |  |  |  |  |  |  |  |  | |  | |  | |  | |  | |  |  |  | |  |  |  |  |  | |  | | | |  |
|  |  |  |  |  |  |  |  |  |  |  | |  | |  | |  | |  | |  |  |  | |  |  |  |  |  | |  | | | |  |
|  |  |  |  |  |  |  |  |  |  |  | |  | |  | |  | |  | |  |  |  | |  |  |  |  |  | |  | | | |  |
|  |  |  |  |  |  |  |  |  |  |  | |  | |  | |  | |  | |  |  |  | |  |  |  |  |  | |  | | | |  |
|  |  |  |  |  |  |  |  |  |  |  | |  | |  | |  | |  | |  |  |  | |  |  |  |  |  | |  | | | |  |
|  |  |  |  |  |  |  |  |  |  |  | |  | |  | |  | |  | |  |  |  | |  |  |  |  |  | |  | | | |  |
|  | | | | | | | | | | | | | | | | | | | | | | | | | | | | |  | |  |  |  |  |
|  |  | | | | | | | | | | | | | | | | | | | | | | | | | | | |  | | |  |  |  |
|  |  | | | | | | | | | | |  | | | | | | | | | | | | | | |  |  | |  | | | | |
|  |  |  |  |  |  | | |  | | |  | |  | |  | |  | |  | | | |  | | | |  |  | |  | | | | |
|  |  |  |  |  |  |  |  |  |  |  | |  | |  | |  | |  | |  |  |  | |  |  |  |  |  | |  | | | |  |
|  |  |  |  |  |  |  |  |  |  |  | |  | |  | |  | |  | |  |  |  | |  |  |  |  |  | |  | | | |  |
|  |  |  |  |  |  |  |  |  |  |  | |  | |  | |  | |  | |  |  |  | |  |  |  |  |  | |  | | | |  |
|  |  |  |  |  |  |  |  |  |  |  | |  | |  | |  | |  | |  |  |  | |  |  |  |  |  | |  | | | |  |
|  |  |  |  |  |  |  |  |  |  |  | |  | |  | |  | |  | |  |  |  | |  |  |  |  |  | |  | | | |  |
|  |  |  |  |  |  |  |  |  |  |  | |  | |  | |  | |  | |  |  |  | |  |  |  |  |  | |  | | | |  |
|  |  |  |  |  |  |  |  |  |  |  | |  | |  | |  | |  | |  |  |  | |  |  |  |  |  | |  | | | |  |
|  |  |  |  |  |  |  |  |  |  |  | |  | |  | |  | |  | |  |  |  | |  |  |  |  |  | |  | | | |  |
|  |  |  |  |  |  |  |  |  |  |  | |  | |  | |  | |  | |  |  |  | |  |  |  |  |  | |  | | | |  |
|  |  |  |  |  |  |  |  |  |  |  | |  | |  | |  | |  | |  |  |  | |  |  |  |  |  | |  | | | |  |
|  | | | | | | | | | | | | | | | | | | | | | | | | | | | | | | | | |  |  |
|  | | | | | | | | | | | | | | | | | | | | | | | | | | | | | | | | | | |
|  | | | | | | | | | | | | | | | | | | | | | | | | | | | | | | | | | | |

|  | Cluster randomised analyses | | | | | | | | | | | | | | | | | | | | | | | | | | | |  | | |  |  |  |
| --- | --- | --- | --- | --- | --- | --- | --- | --- | --- | --- | --- | --- | --- | --- | --- | --- | --- | --- | --- | --- | --- | --- | --- | --- | --- | --- | --- | --- | --- | --- | --- | --- | --- | --- |
|  | **Control group** | | | | | | | | | | | **Strategy group** | | | | | | | | | | | | | | |  |  | |  | | | | |
|  | *No.* |  | *(%)* |  | *Mean ± SD* | | | *Median (IQR)* | | | *No.* | |  | | *(%)* | |  | | *Mean ± SD* | | | | *Median (IQR)* | | | | *Mean difference^a^* | *Confidence interval^b^* | | *P-Value^c^* | | | | |
| Weight evolution between D0 and D7 | 324 |  | (68.6%) |  | 2.3 | ± | 7.2 | 2.0 |  | (-2.0-7.0) | | 310 | |  | | (71.6%) | |  | | 1.2 | ± | 6.1 | | 1.0 |  | (-2.0-4.0) | -1.1 | (-2.7 ; 0.5) | | 0.70 | | | |  |
| Weight evolution between D0 and D14 | 152 |  | (32.2%) |  | -0.5 | ± | 8.3 | -1.0 |  | (-4.0-4.0) | | 142 | |  | | (32.8%) | |  | | 0.4 | ± | 7.3 | | 0.0 |  | (-4.0-4.0) | 0.8 | (-2.0 ; 3.6) | | >0.99 | | | |  |
| Average fluid intake from D0 to D7 (mL) | 472 |  | (100%) |  | 2697.2 | ± | 1024.5 | 2547.6 |  | (2031.6-3194.8) | | 432 | |  | | (99.8%) | |  | | 2531.2 | ± | 897.9 | | 2400.6 |  | (1900.3-3040.1) | -166.0 | (-361.1 ; 29.0) | | 0.20 | | | |  |
| Average fluid output from D0 to D7 (mL) | 472 |  | (100%) |  | 1871.6 | ± | 821.7 | 1755.9 |  | (1387.8-2321.9) | | 433 | |  | | (100%) | |  | | 1883.0 | ± | 730.4 | | 1823.1 |  | (1368.1-2266.9) | 11.3 | (-145.9 ; 168.6) | | >0.99 | | | |  |
| Average fluid balance from D0 to D7 (mL) | 472 |  | (100%) |  | 825.6 | ± | 1070.9 | 627.7 |  | (176.6-1370.4) | | 433 | |  | | (100%) | |  | | 642.3 | ± | 844.6 | | 541.0 |  | (133.3-1051.7) | -183.2 | (-378.8 ; 12.3) | | 0.09 | | | |  |
| Average fluid intake from D0 to D14 (mL) | 472 |  | (100%) |  | 2563.5 | ± | 936.3 | 2437.6 |  | (1940.0-3057.4) | | 433 | |  | | (100%) | |  | | 2445.0 | ± | 857.3 | | 2364.6 |  | (1820.9-2896.1) | -118.5 | (-300.0 ; 62.9) | | 0.96 | | | |  |
| Average fluid output from D0 to D14 (mL) | 472 |  | (100%) |  | 1893.1 | ± | 780.2 | 1813.0 |  | (1426.3-2345.1) | | 433 | |  | | (100%) | |  | | 1838.7 | ± | 680.1 | | 1805.0 |  | (1392.7-2243.6) | -54.4 | (-202.5 ; 93.7) | | >0.99 | | | |  |
| Average fluid balance from D0 to D14 (mL) | 472 |  | (100%) |  | 670.4 | ± | 927.1 | 514.7 |  | (159.7-1024.6) | | 433 | |  | | (100%) | |  | | 606.3 | ± | 748.4 | | 551.8 |  | (140.6-953.6) | -64.1 | (-234.9 ; 106.6) | | >0.99 | | | |  |
| Total dose of diuretics from D0 to D14 (mg) | 328 |  | (69.5%) |  | 814.3 | ± | 1655.1 | 305.0 |  | (80.0-726.5) | | 314 | |  | | (72.5%) | |  | | 455.6 | ± | 741.5 | | 220.0 |  | (80.0-520.0) | -358.7 | (-668.3 ; -49.2) | | >0.99 | | | |  |
| Total dose of albumin 20% from D0 to D14 (mL) | 47 |  | (10%) |  | 689.4 | ± | 783.6 | 400.0 |  | (300.0-1000.0) | | 60 | |  | | (13.9%) | |  | | 863.3 | ± | 860.4 | | 550.0 |  | (300.0-1000.0) | 174.0 | (-325.5 ; 673.4) | | >0.99 | | | |  |
|  | | | | | | | | | | | | | | | | | | | | | | | | | | | | |  | |  |  |  |  |
|  | Randomised before-and-after analyses | | | | | | | | | | | | | | | | | | | | | | | | | | | |  | | |  |  |  |
|  | **Control group** | | | | | | | | | | | **Strategy group** | | | | | | | | | | | | | | |  |  | |  | | | | |
|  | *No.* |  | *(%)* |  | *Mean ± SD* | | | *Median (IQR)* | | | *No.* | |  | | *(%)* | |  | | *Mean ± SD* | | | | *Median (IQR)* | | | | *Mean difference^a^* | *Confidence interval^b^* | | *P-Value^c^* | | | | |
| Weight evolution between D0 and D7 | 467 |  | (65%) |  | 2.6 | ± | 7.2 | 2.0 |  | (-1.0-7.0) | | 465 | |  | | (72.3%) | |  | | 1.7 | ± | 6.1 | | 1.0 |  | (-2.0-5.0) | -0.9 | (-2.2 ; 0.5) | | 0.93 | | | |  |
| Weight evolution between D0 and D14 | 219 |  | (30.5%) |  | -0.0 | ± | 8.3 | -1.0 |  | (-4.0-5.0) | | 211 | |  | | (32.8%) | |  | | 0.4 | ± | 7.4 | | 0.0 |  | (-4.0-4.0) | 0.4 | (-1.9 ; 2.7) | | >0.99 | | | |  |
| Average fluid intake from D0 to D7 (mL) | 718 |  | (100%) |  | 2614.2 | ± | 950.0 | 2504.4 |  | (1985.0-3071.8) | | 642 | |  | | (99.8%) | |  | | 2487.6 | ± | 886.1 | | 2380.4 |  | (1847.3-3003.6) | -126.6 | (-278.0 ; 24.9) | | 0.23 | | | |  |
| Average fluid output from D0 to D7 (mL) | 718 |  | (100%) |  | 1744.8 | ± | 788.4 | 1684.6 |  | (1260.0-2163.8) | | 643 | |  | | (100%) | |  | | 1856.4 | ± | 706.9 | | 1800.0 |  | (1379.4-2256.3) | 111.6 | (-11.9 ; 235.1) | | 0.13 | | | |  |
| Average fluid balance from D0 to D7 (mL) | 718 |  | (100%) |  | 869.4 | ± | 1039.6 | 717.6 |  | (211.8-1440.8) | | 643 | |  | | (100%) | |  | | 627.4 | ± | 866.3 | | 544.0 |  | (51.7-1096.9) | -242.0 | (-400.2 ; -83.9) | | <0.001 | | | |  |
| Average fluid intake from D0 to D14 (mL) | 718 |  | (100%) |  | 2481.7 | ± | 863.0 | 2374.1 |  | (1917.5-2928.4) | | 643 | |  | | (100%) | |  | | 2377.8 | ± | 845.4 | | 2298.8 |  | (1774.5-2819.7) | -103.8 | (-244.4 ; 36.7) | | 0.51 | | | |  |
| Average fluid output from D0 to D14 (mL) | 718 |  | (100%) |  | 1763.4 | ± | 752.3 | 1720.5 |  | (1297.4-2151.0) | | 643 | |  | | (100%) | |  | | 1823.3 | ± | 654.7 | | 1782.7 |  | (1400.0-2203.3) | 59.9 | (-56.5 ; 176.4) | | >0.99 | | | |  |
| Average fluid balance from D0 to D14 (mL) | 718 |  | (100%) |  | 718.3 | ± | 900.0 | 599.5 |  | (198.7-1122.8) | | 643 | |  | | (100%) | |  | | 554.5 | ± | 765.1 | | 518.8 |  | (51.7-951.6) | -163.8 | (-301.8 ; -25.8) | | 0.007 | | | |  |
| Total dose of diuretics from D0 to D14 (mg) | 475 |  | (66.2%) |  | 669.2 | ± | 1432.6 | 220.0 |  | (80.0-620.0) | | 478 | |  | | (74.3%) | |  | | 630.8 | ± | 1297.8 | | 233.0 |  | (80.0-590.0) | -38.4 | (-306.8 ; 230.0) | | >0.99 | | | |  |
| Total dose of albumin 20% from D0 to D14 (mL) | 111 |  | (15.5%) |  | 621.2 | ± | 592.8 | 400.0 |  | (300.0-800.0) | | 91 | |  | | (14.2%) | |  | | 763.7 | ± | 755.8 | | 500.0 |  | (300.0-1000.0) | 142.6 | (-148.0 ; 433.2) | | >0.99 | | | |  |
| ^a^ Mean difference was assessed with the difference between Strategy and Control groups | | | | | | | | | | | | | | | | | | | | | | | | | | | | | | | | |  |  |
| ^b^ Confidence intervals (CIs) were obtained with Bonferroni adjustment : Alpha = 0.05/20=0.0025 | | | | | | | | | | | | | | | | | | | | | | | | | | | | | | | | | | |
| ^c^ P-values were transformed using Bonferroni adjustment: multiplied by 20 | | | | | | | | | | | | | | | | | | | | | | | | | | | | | | | | | | |

## Table S4 – Primary, Secondary, and Safety Outcomes in randomised before-and-after analyses of the POINCARE-2 trial

|  |  |  |  |  | |  | |  | |
| --- | --- | --- | --- | --- | --- | --- | --- | --- | --- |
|  |  |  |  |  |  |  |  | |  |
|  |  |  |  |  |  |  |  | |  |
|  |  |  |  |  |  |  |  | |  |
|  |  |  |  |  |  |  |  | |  |
|  |  |  |  |  |  |  |  | |  |
|  |  |  |  |  |  |  |  | |  |
|  |  |  |  |  |  |  |  | |  |
|  |  |  |  |  |  |  |  | |  |
|  |  |  |  |  |  |  |  | |  |
|  |  |  |  |  |  |  |  | |  |
|  |  |  |  |  |  |  |  | |  |
|  |  |  |  |  |  |  |  | |  |
|  |  |  |  |  |  |  |  | |  |
|  |  |  |  |  |  |  |  | |  |
|  |  |  |  |  |  |  |  | |  |
|  |  |  |  |  |  |  |  | |  |
|  |  |  |  |  |  |  |  | |  |
|  |  |  |  |  |  |  |  | |  |
|  |  |  |  |  |  |  |  | |  |
|  | | | | | | | | | |
|  | | | | | | | | | |
|  | | | | | | | | | |
|  | | | | | | | | | |
|  | | | | | | | | | |
|  | | | | | | | | | |
|  | | | | | | | | | |
|  | | | | | | | | | |


|  | Control group | Strategy group |  | Crude effect of strategy | | | | |  | Adjusted effect of strategy^a^ | | | | |
| --- | --- | --- | --- | --- | --- | --- | --- | --- | --- | --- | --- | --- | --- | --- |
|  | % [95% CI]^b^ | % [95% CI]^b^ |  | model (part) | | exp(parameter) [95% CI] | | P-value |  | model (part) | | exp(parameter) [95% CI] | | P-value |
|  | median (IQR)^c^ | median (IQR)^c^ |  |  |  |  |  |  |  |  |  |  |  |  |
| *Primary outcome* |  |  |  |  |  |  |  |  |  |  |  |  |  |  |
| 60-day mortality rate^b^ | 34.5 [31.1-38.0] | 30.3 [26.8-33.9] |  | MP |  | RR | 0.88 [0.70-1.10]^d^ | >0.99^d^ |  | MP |  | RR | 0.94 [0.77-1.14]^d^ | >0.99^d^ |
| *Secondary outcomes* |  |  |  |  |  |  |  |  |  |  |  |  |  |  |
| 28-day mortality rate^b^ | 27.3 [24.0-30.6] | 23.3 [20.0-26.6] |  | MP |  | RR | 0.86 [0.66-1.11]^d^ | >0.99^d^ |  | MP |  | RR | 0.92 [0.71-1.19]^d^ | >0.99^d^ |
| In-hospital mortality rate^b^ | 33.0 [29.6-36.5] | 29.2 [25.7-32.8] |  | MP |  | RR | 0.89 [0.70-1.11]^d^ | >0.99^d^ |  | MP |  | RR | 0.95 [0.78-1.16]^d^ | >0.99^d^ |
| Mechanical ventilator-free days^c^ | 8.0 (0.0-17.0) | 10.0 (0.0-17.0) |  | ZINB | (NB) | MF | 1.02 [0.94-1.11]^d^ | >0.99^d^ |  | ZINB | (NB) | MF | 1.02 [0.94-1.11]^d^ | >0.99^d^ |
|  |  |  |  |  | (ZI) | OR | 0.83 [0.56-1.22]^d^ | >0.99^d^ |  |  | (ZI) | OR | 0.89 [0.60-1.32]^d^ | >0.99^d^ |
| Vasopressor-free days^c^ | 16.5 (7.0-24.0) | 16.0 (8.0-25.0) |  | ZINB | (NB) | MF | 1.00 [0.95-1.05]^d^ | >0.99^d^ |  | ZINB | (NB) | MF | 0.99 [0.93-1.05]^d^ | >0.99^d^ |
|  |  |  |  |  | (ZI) | OR | 0.89 [0.47-1.67]^d^ | >0.99^d^ |  |  | (ZI) | OR | 1.05 [0.58-1.89]^d^ | >0.99^d^ |
| Renal replacement therapy-free days^c^ | 22.5 (11.0-45.0) | 22.0 (11.0-47.0) |  | ZIPoi | (Poi) | MF | 0.996 [0.96-1.03]^d^ | >0.99^d^ |  | ZIPoi | (Poi) | MF | 0.99 [0.96-1.02]^d^ | >0.99^d^ |
|  |  |  |  |  | (ZI) | OR | 1.11 [0.37-3.34]^d^ | >0.99^d^ |  |  | (ZI) | OR | 1.62 [0.55-4.76]^d^ | >0.99^d^ |
| *Safety Outcomes* |  |  |  |  |  |  |  |  |  |  |  |  |  |  |
| Arterial hypotension^b^ | 75.1 [71.9-78.2] | 75.4 [72.1-78.8] |  | MP |  | RR | 1.02 [0.95-1.08] | 0.65 |  | MP |  | RR | 1.03 [0.97-1.10] | 0.30 |
| At least one episode of hypernatremia > 155 mmol/l^b^ | 2.4 [1.3-3.5] | 4.7 [3.0-6.3] |  | LB |  | RR | 1.93 [1.07-3.48] | 0.03 |  | LB |  | RR | 1.85 [1.03-3.33] | 0.04 |
| At least one episode of hypokalaemia < 2.8 mmol/l^b^ | 7.4 [5.5-9.3] | 7.6 [5.6-9.7] |  | LB |  | RR | 1.01 [0.70-1.47] | 0.95 |  | LB |  | RR | 1.06 [0.73-1.54] | 0.76 |
| Renal damage^b,e^ | 19.4 [16.5-22.4] | 21.7 [18.5-24.9] |  | LB |  | RR | 1.13 [0.90-1.41] | 0.30 |  | LB |  | RR | 1.12 [0.90-1.38] | 0.32 |
| Myocardial infarction^b^ | 0.6 [0.0 – 1.1] | 0.8 [0.1-1.5] |  | LB |  | RR | 1.78 [0.46-6.91] | 0.40 |  | LB |  | RR | 1.68 [0.43-6.58] | 0.46 |
| Mesenteric ischaemia^b^ | 0.6 [0.0-1.1] | 0.2 [0.0-0.5] |  | LB |  | RR | 0.31 [0.03-2.78] | 0.29 |  | LB |  | RR | 0.42 [0.04-4.01] | 0.45 |
| CI denotes confidence interval, IQR interquartile range, MP mixed modified Poisson, RR risk ratio, ZINB zero-inflated negative binomial mixed, NB negative binomial part, MF multiplicative factor, ZI zero-inflated part, ZIPoi zero-inflated Poisson mixed, Poi Poisson part and LB log binomial mixed.  ^a^ Adjusted for the following baseline characteristics: age, main cause of admission (recoded as CNS injury vs. other causes of admission), McCabe score, SAPS II at day 1.  ^b^ Binary outcomes : log-binomial (modified Poisson) mixed models were used and “exp(parameter)” are estimations of RR.  ^c^ Numeric outcomes : modeled with zero-inflated negative binomial (Poisson) mixed models which combine a logistic regression and a negative binomial (Poisson respectively) model and “exp(parameter)” are odds ratios of excess of zeros for the zero-inflated part (logistic) and multiplicative factor for the counting part (negative binomial/Poisson). ^d^ Bonferroni correction was used: Confidence intervals according to α=5/22=0.23% and p-values by a multiplying a coefficient of 22.  ^e^ Renal damage was defined by worsening RIFLE between Day3 and Day14 as compared with the higher RIFLE observed on Day1 or Day2. For renal damage, analyses were conducted within the subsample of at-risk patients (i.e., in 883/905 patients not classified as RIFLE “End Stage Kidney Disease” at Day 2) | | | | | | | | | | | | | | |

## Table S5 – Intra-cluster, intra-period and intra-cluster-period correlation for 60-day mortality

| Correlation | Coefficient |
| --- | --- |
| Intra-cluster | 0.013 |
| Intra-period | 0.000 |
| Intra-cluster-period | 0.015 |
|  | |

# FIGURES

Figure S1 –POINCARE-2 strategy of fluid balance control in critically ill patients (reproduced from 19)
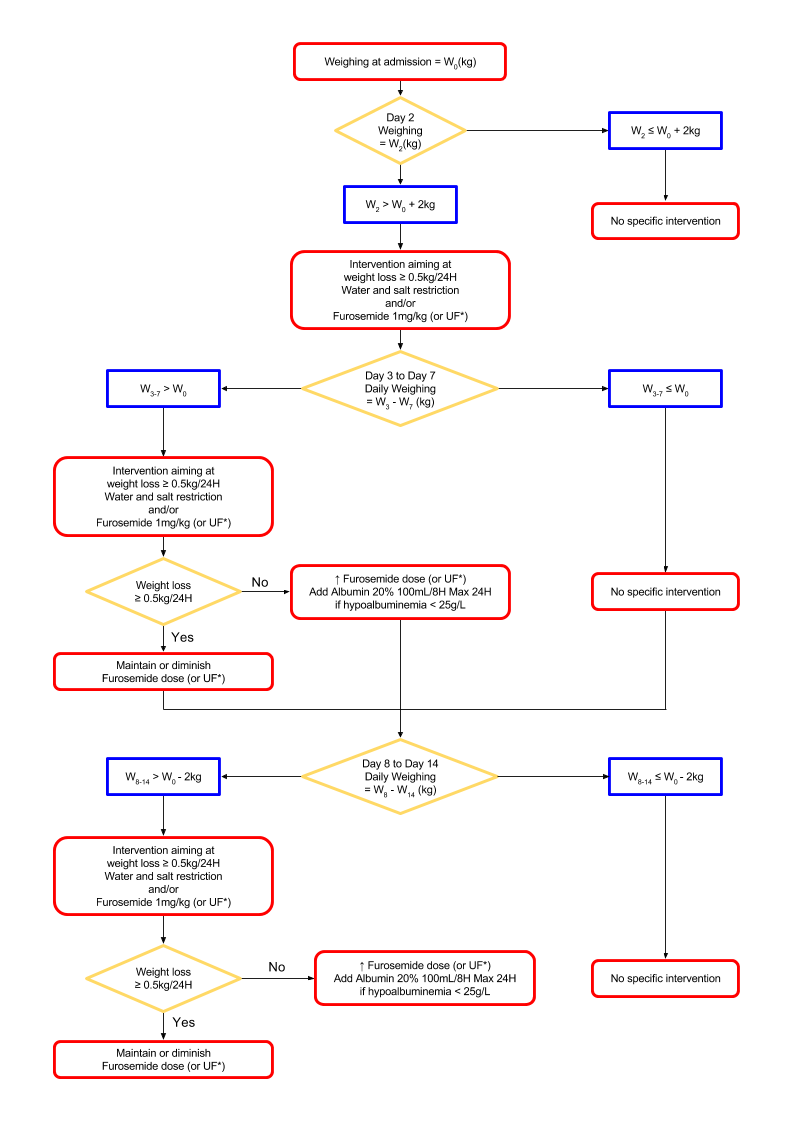


Note: * Ultrafiltration in case of renal replacement therapy

## Figure S2 – Evolution of the average weight from Day 0 to Day 14 in patients in randomized before-and-after analyses of the POINCARE-2 trial (with 95% confidence interval of mean and sample size as point label)


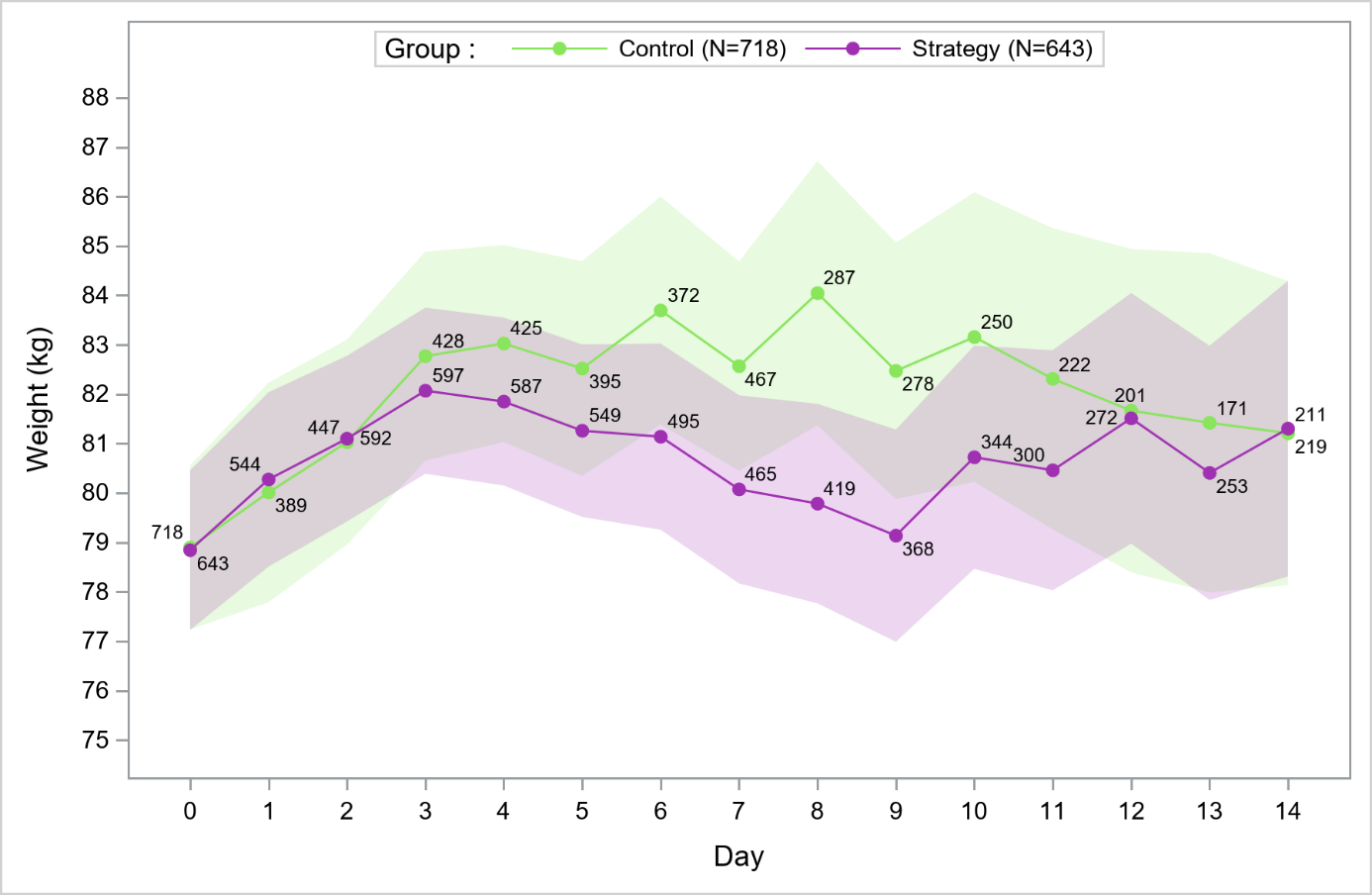


Notes: Using a mixed model entering ICU and patient as random effects, and the time-period (defined as ≤ 2 days vs. > 2 days) interaction with the linear time as a fixed effect, we found no difference in trend between the 2 groups during the second time period (> 2 days): p=0.84.

## Figure S3 – Smoothed cumulated fluid balance evolution from Day 0 to Day 14 in patients in cluster randomised analyses of the POINCARE-2 trial (with 95% confidence interval of mean prediction)


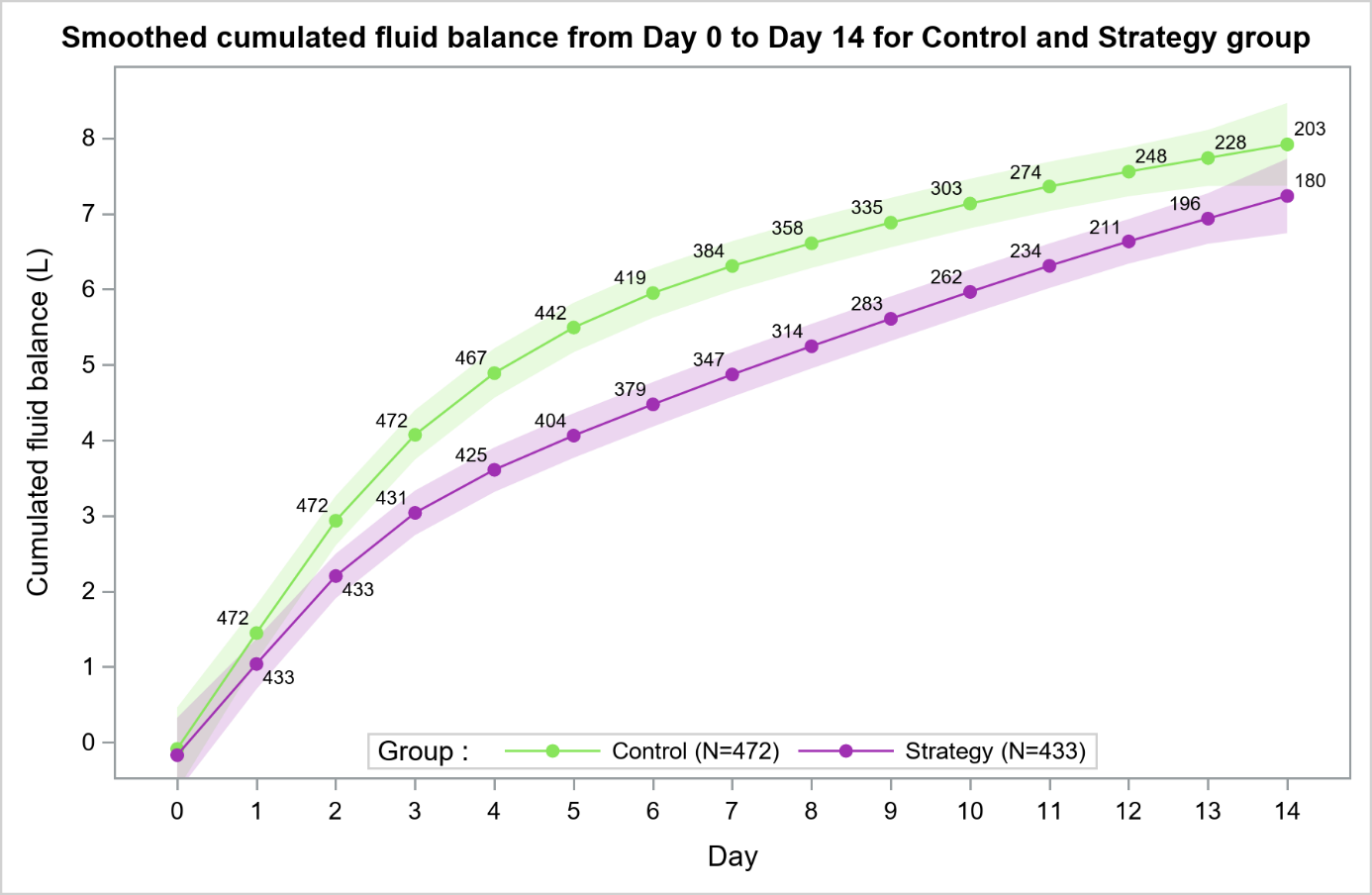


## Figure S4 – Smoothed cumulated fluid balance evolution from Day 0 to Day 14 in patients in randomised before-and-after analyses of the POINCARE-2 trial (with 95% confidence interval of mean prediction)


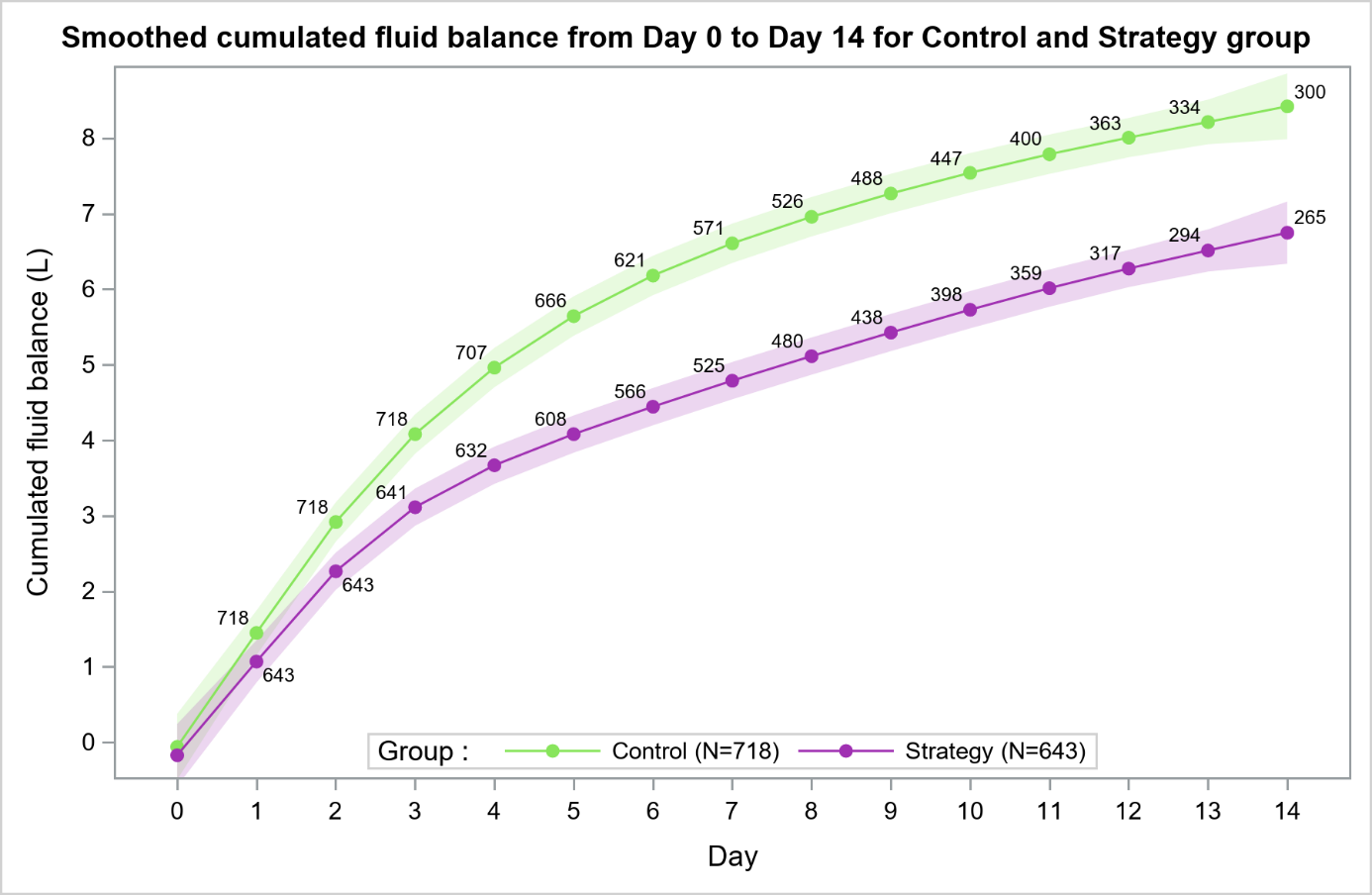


## Figure S5 – Survival of patients included in cluster randomised analyses of the POINCARE-2 trial


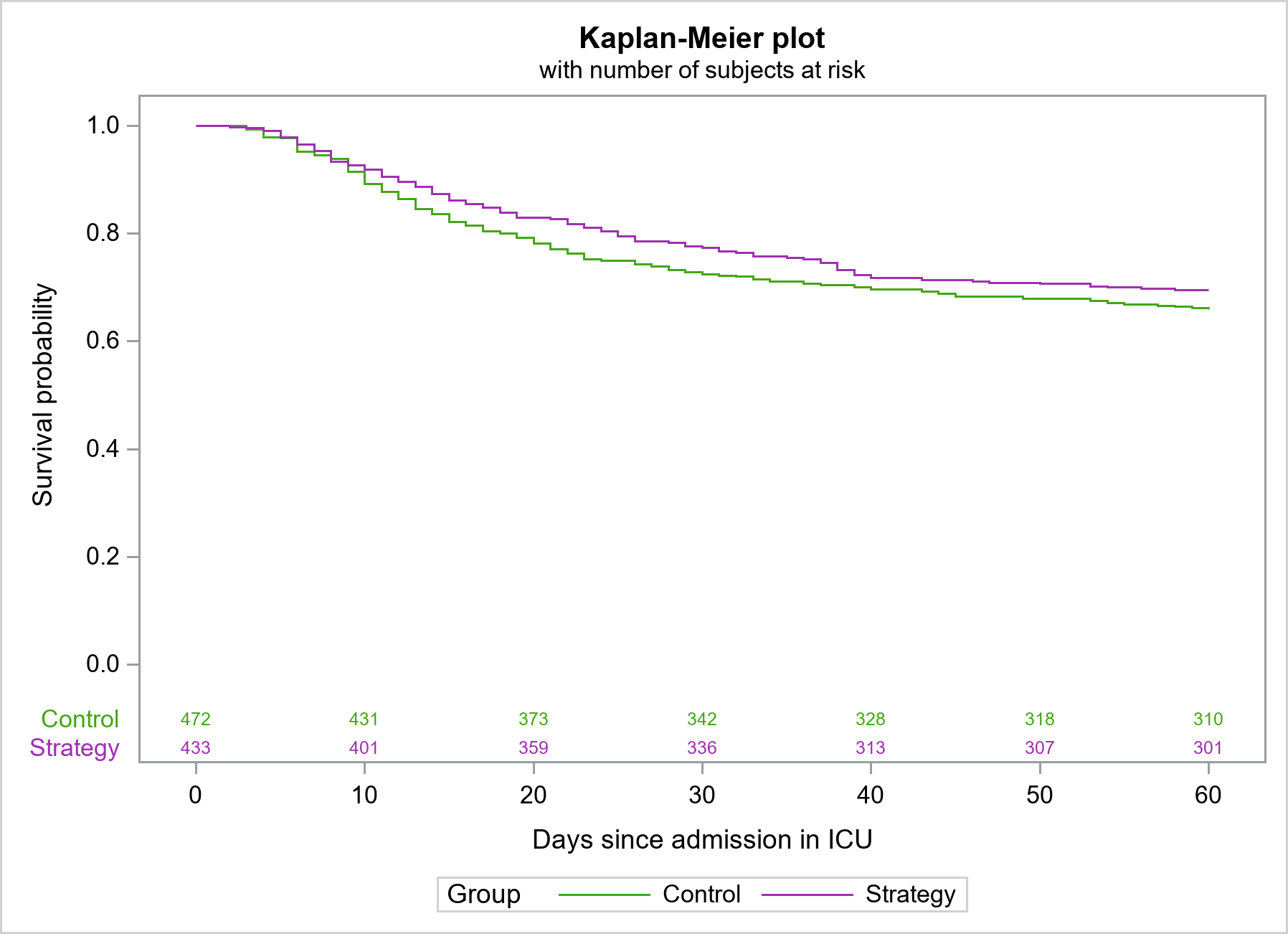


## Figure S6 – Survival of patients included in randomised before-and-after analyses of the POINCARE-2 trial


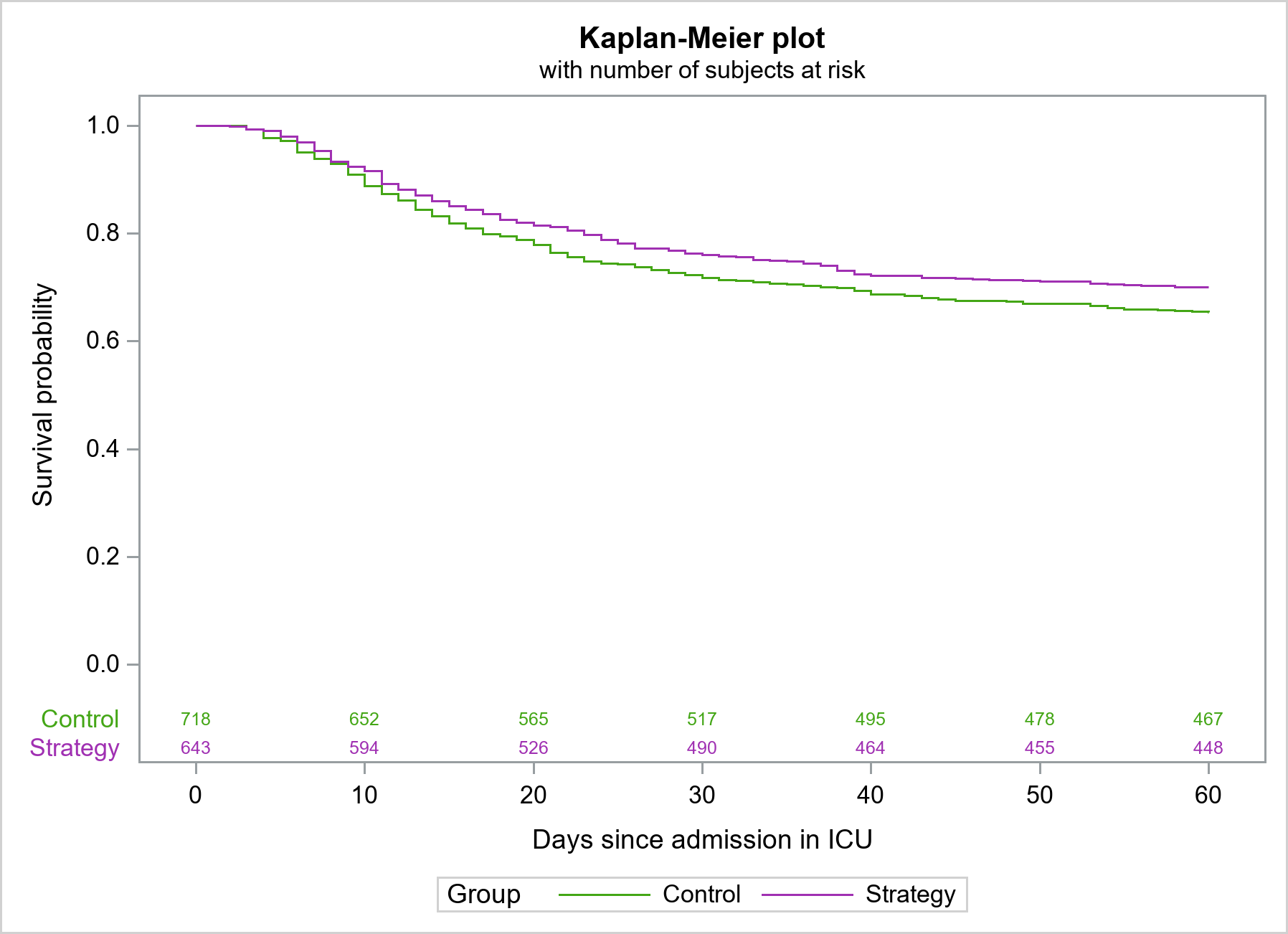

Supplement: Supplementary file 1 — Additional file 1: Fig. S1. POINCARE-2 strategy of fluid balance control in critically ill patients (reproduced from 19). Table S1. Baseline characteristics of patients in randomized before-and-after analyses of the POINCARE-2 trial. Table S2. Description of missing data at baseline for patients in cluster-randomized (CRA) and randomized before-and-after (RBAA) analyses of the POINCARE-2 trial. Table S3. Adherence to the POINCARE-2 strategy in cluster-randomized and randomized before-and-after analyses. Fig. S2. Evolution of the average weight from Day 0 to Day 14 in patients in randomized before-and-after analyses of the POINCARE-2 trial (with 95% confidence interval of mean and sample size as point label). Fig. S3. Smoothed cumulated fluid balance evolution from Day 0 to Day 14 in patients in cluster-randomized analyses of the POINCARE-2 trial (with 95% confidence interval of mean prediction). Table S4. Primary, Secondary, and Safety Outcomes in randomized before-and-after analyses of the POINCARE-2 trial. Table S5. Intra-cluster, intra-period and intra-cluster-period correlation for 60-day mortality. Fig. S4. Smoothed cumulated fluid balance evolution from Day 0 to Day 14 in patients in randomized before-and-after analyses of the POINCARE-2 trial (with 95% confidence interval of mean prediction). Fig. S5. Survival of patients included in cluster-randomized analyses of the POINCARE-2 trial. Fig. S6. Survival of patients included in randomized before-and-after analyses of the POINCARE-2 trial. [file 13054_2023_4357_MOESM1_ESM.docx]
